# Multi-Epitope Vaccine Design Using an Immunoinformatic Approach for SARS-CoV-2

**DOI:** 10.3390/pathogens10060737

**Published:** 2021-06-11

**Authors:** Ye Feng, Haiping Jiang, Min Qiu, Liang Liu, Shengmei Zou, Yun Li, Qianpeng Guo, Ning Han, Yingqiang Sun, Kui Wang, Lantian Lu, Xinlei Zhuang, Shanshan Zhang, Shuqing Chen, Fan Mo

**Affiliations:** 1 Sir Run Run Shaw Hospital, Zhejiang University School of Medicine, Hangzhou 310001, China; pandafengye@zju.edu.cn (Y.F.); 21818662@zju.edu.cn (S.Z.); 2 Institute for Translational Medicine, Zhejiang University School of Medicine, Hangzhou 310002, China; 3 The First Affiliated Hospital, Zhejiang University School of Medicine, Hangzhou 310007, China; jianghaiping@zju.edu.cn; 4 Hangzhou Neoantigen Therapeutics Co., Ltd., Hangzhou 310058, China; qium@neoantigen.cn (M.Q.); liul@neoantigen.cn (L.L.); guoqp@neoantigen.cn (Q.G.); hann@neoantigen.cn (N.H.); sunyq@neoantigen.cn (Y.S.); wangk@neoantigen.cn (K.W.); l.lu@uq.edu.au (L.L.); zhangss@neoantigen.cn (S.Z.); 5 Zhejiang Forest Resources Monitoring Center, Hangzhou 310020, China; lz123698@foxmail.com; 6 College of Pharmaceutical Sciences, Zhejiang University, Hangzhou 310058, China; 21819022@zju.edu.cn; 7 Zhejiang California International Nanosystems Institute, Zhejiang University, Hangzhou 310058, China; 8 Vancouver Prostate Centre, University of British Columbia, Vancouver, BC V6H 3Z6, Canada; 9 Hangzhou AI-Force Therapeutics Co., Ltd., Hangzhou 310000, China

**Keywords:** COVID-19, SARS-CoV-2, epitope, immunoinformatics, peptide, vaccine

## Abstract

Through 4 June 2021, COVID-19 has caused over 172.84 million cases of infection and 3.71 million deaths worldwide. Due to its rapid dissemination and high mutation rate, it is essential to develop a vaccine harboring multiple epitopes and efficacious against multiple variants to prevent the immune escape of SARS-CoV-2. An in silico approach based on the viral genome was applied to identify 19 high-immunogenic B-cell epitopes and 499 human leukocyte antigen (HLA)-restricted T-cell epitopes. Thirty multi-epitope peptide vaccines were designed by iNeo-Suite and manufactured by solid-phase synthesis. Docking analysis confirmed stable hydrogen bonds of epitopes with their corresponding HLA alleles. When four peptide candidates derived from the spike protein of SARS-CoV-2 were selected to immunize mice, a significantly larger amount of total IgG in serum, as well as an increase of CD19+ cells in the inguinal lymph nodes, were observed in the peptide-immunized mice compared to the control. The ratios of IFN-γ-secreting lymphocytes in CD4+ or CD8+ T-cells in the peptide-immunized mice were higher than those in the control mice. There were also a larger number of IFN-γ-secreting T-cells in the spleens of peptide-immunized mice. The peptide vaccines in this study successfully elicited antigen-specific humoral and cellular immune responses in mice. To further validate the safety and efficacy of this vaccine, animal studies using a primate model, as well as clinical trials in humans, are required.

## 1. Introduction

In December 2019, SARS-CoV-2 caused an outbreak of viral lung infections in Wuhan City, Hubei Province, China, and later infected people in 213 countries worldwide [1,2,3,4,5]. A genome sequence comparison of SARS-CoV-2 with SARS-CoV and bat coronaviruses showed 79.5% and 96% similarities at the nucleotide level, respectively [6], suggesting its probable origin in bats [7].

Up until now, several therapeutics and vaccines have been used under Emergency Use Authorization for COVID-19 [8]. However, the majority of disease control has extensively relied on the rapid detection and isolation of symptomatic cases [9]. Therefore, a safe and efficacious vaccine is urgently needed. In comparison with traditional vaccine-developing approaches, which require the isolation, inactivation and injection of pathogens (or portions of them), computation-based methods generally start by analyzing the genomes of pathogens and can speed up the entire process for vaccine development, due to rapid progress. The complete genome of SARS-CoV-2 encodes the spike protein, membrane protein, envelope protein and nucleocapsid protein, as well as other replication- and transcription-related enzymes. Due to the lack of a repair mechanism in the RNA virus replicase complex, mutations are prone to occur during replication of SARS-CoV-2. The 4% nucleotide difference between the viruses isolated from Rhinolophus and those from humans suggests that SARS-CoV-2 mutates rapidly to achieve host conversion [10,11]. Similar to SARS-CoV, SARS-CoV-2 uses its receptor binding domain (RBD) on the spike protein to bind to a host cell’s angiotensin-converting enzyme 2 (ACE2) [6,9,12,13]. 10- to 20-fold higher binding affinity to ACE2 was observed for SARS-CoV-2 compared to SARS-CoV [14]. Consequently, vaccines against SARS-CoV-2 can be developed targeting its structural proteins and in particular the RBD region, following the strategy for SARS-CoV vaccine development [12,15,16,17,18].

An ideal vaccine containing both B-cell and T-cell epitopes can elicit both humoral and cellular immune responses against a specific pathogen in an efficient manner [19]. Since the first peptide vaccine against the virus that caused foot-and-mouth disease was developed [20], the subsequent establishment of the peptide synthesis method by Lerner et al. [21] along with the strategy of combining both T-cell and B-cell epitopes in the design of a peptide vaccine has accelerated vaccine development. In the present study, we followed this in silico approach to identify potential B-cell and T-cell epitope(s) from the spike, envelope and membrane proteins of SARS-CoV-2. Next, we selected a few candidate peptides to immunize mice. As a result, these peptides successfully elicited specific humoral and cellular immune responses, showing their real potential to combat SARS-CoV-2.

## 2. Results

### 2.1. Prediction of B-Cell Epitopes

**Figure 1:**
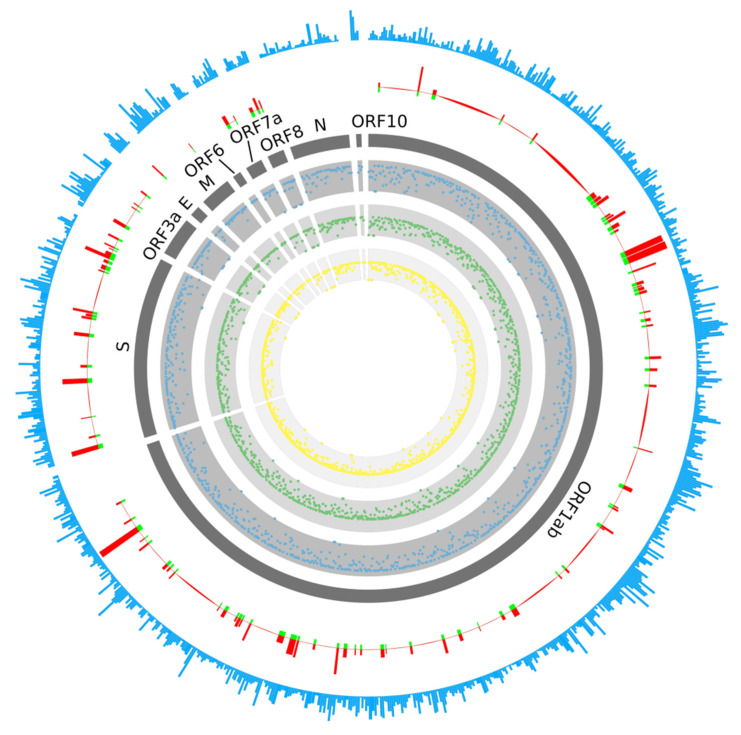
Distribution of B-cell and T-cell epitopes. The outermost circle (light blue) stands for the T-cell epitope count. The second-outermost circle stands for Emini (in red) and Kolaskar (in green) scores, used to evaluate the B-cell epitopes (these methods were based on linear B-cell epitope sequences). The 3rd circle displays the names of different viral proteins. The 4th–6th circles stand for HLA-A (in blue), HLA-B (in green) and HLA-C (in yellow) scores, respectively; the points closer to the center indicate a lower score.

**Figure 2:**
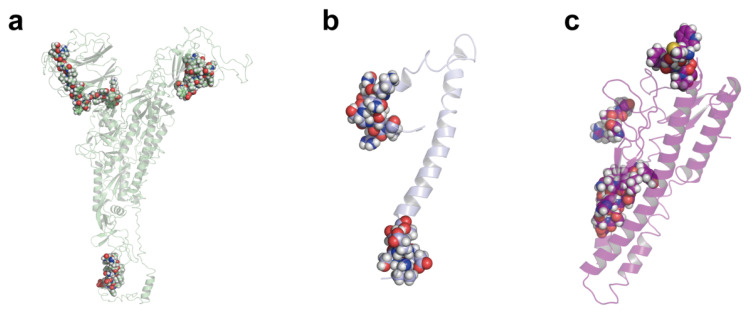
Locations of the recognized B-cell epitopes on the viral spike protein (**a**), envelope protein (**b**) and membrane protein (**c**). The transparent cartoon models show the predicted 3D structures; the colorful spheres suggest the positions of the recognized epitopes.

During viral infection, B-cells take in viral epitopes to recognize viruses, thereby activating defense responses. Recognition of B-cell epitopes depends on the antigenicity, surface accessibility and predictions of linear epitopes [22,23]. In this study, a total of 61 B-cell epitopes were predicted, which seemed to preferentially locate within certain regions of the gene (Figure 1 and Figure 2, Table S1). Only 19 out of 61 epitopes were predicted to be exposed on the surface of the virion and had high antigenicity scores, indicating their potential for initiating strong immune responses. Therefore, they were considered promising vaccine candidates for targeting B-cells. Among the 19 epitopes, 17 were longer than 14 amino acids, were located in the spike protein containing RBD and functioned in host cell binding (Table 1). The average Emini score for the 19 epitopes was 2.744, and the average Kolaskar (antigenicity) score was 1.015. Two epitopes were located within the RBD region, while the one with the higher Kolaskar score (1.059), 1052-FPQSAPH-1058, was located at position 1052aa of the spike protein.

### 2.2. Prediction of T-Cell Epitopes

T-cell immune response is considered to be a longer-lasting response compared to B-cell immune response, wherein the antigen might easily escape the antibody memory response [24]. Moreover, the CD4+ and CD8+ T-cell responses play a major role in antiviral immunity. Therefore, it is important to design vaccines that can induce T-cell immune response [25]. A total of 499 T-cell epitopes were predicted on the spike protein (378 epitopes), membrane protein (90 epitopes) and envelope protein (31 epitopes) of SARS-CoV-2, respectively; 48 of the 378 epitopes for the spike protein were located in the RBD region (Figure 1, Table 2 and Table S2). There was no preference in certain genes or regions for T-cell epitope generation; no biased distribution of T-cell epitopes among HLA types was observed, either. Among all T-cell epitopes, epitope 869-MIAQYTSAL-877 on the spike protein was predicted to be able to bind to 17 HLA alleles. Most of the HLA alleles included in this study were covered by these candidate peptides, suggesting wide population coverage.

In terms of the distribution of the predicted epitopes against different HLA haplotypes, no significant differences were observed. There were 287, 208 and 195 epitopes predicted to be able to bind to HLA-A, HLA-B and HLA-C haplotypes, respectively. For the five most popular HLA types (HLA-A*11:01, HLA-A*24:02, HLA-C*07:02, HLA-A*02:01 and HLA-B*46:01), the counts for epitopes with a binding affinity were 51, 49, 115, 48 and 58.

### 2.3. Multi-Epitope Vaccine Design

Based on the 19 B-cell epitopes and their 121 adjacent T-cell epitopes, 17 candidate vaccine peptides, containing both B-cell and T-cell epitopes, were generated by our in-house software, iNeo-Design. Most of the 17 candidate vaccine peptides contained one B-cell epitope, except for AVEQDKNTQEVFAQVKQIYKTPPIKDFGG, which included two B-cell epitopes and eight T-cell epitopes and AKNLNESLIDLQELGKYEQYIKWPWYIWKK, which included two B-cell epitopes and 6 T-cell epitopes. By comparison, the vaccine peptide FKNLREFVFKNIDGYFKIYSKHTPINLV had the largest count of T-cell epitopes, whereas SYGFQPTNGVGYQPYRVVVLSFELLHAPAT showed the highest HLA score, indicating their wide population coverage and promising efficacy.

In addition to the vaccine candidates containing both B-cell and T-cell epitopes, we also analyzed all 499 core T-cell epitopes to generate another 102 candidate peptides that contained T-cell epitopes only. Based on both the epitope counts and HLA scores, we eventually selected 13 peptides.

Taken together, a total of 30 peptides were designed as vaccine candidates (Table 3). Twenty-six (86.67%) of them were derived from the spike protein, two (6.67%) from the membrane protein and two (6.67%) from the envelope protein of SARS-CoV-2. Five (16.67%) peptides were located in the RBD region, indicating their likelihood of inducing the production of neutralizing antibodies. The 30 vaccine peptides targeting all structural proteins may induce immune responses against SARS-CoV-2, in theory. The multi-peptide strategy we applied here better fit the genetic variability of the human immune system and reduced the risk of immune escape due to viral genomic mutation [26].

### 2.4. Interaction of Predicted Peptides with HLA Alleles

**Figure 3:**
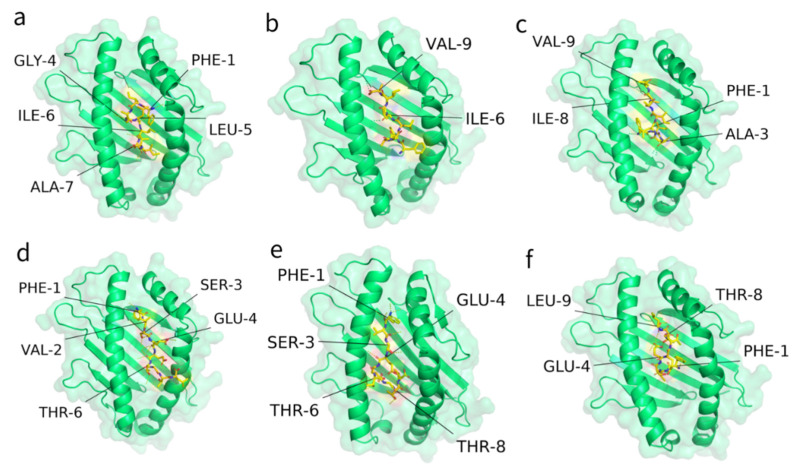
Interactions between the predicted epitopes (yellow sticks) and different HLA alleles (green cartoons). The dotted lines between peptides and HLA are their binding sites. Amino acids are labeled adjacent to the binding sites. Table 4 summarizes the detailed docking information of panels (**a**–**f**).

To further inspect the binding stability of T-cell epitopes with HLA alleles, the T-cell epitopes involved in the above-designed vaccine peptides were selected to conduct an interaction analysis. Figure 3 illustrates the docking results against the most popular HLA types for the two epitopes from vaccine peptides 25 and 27 (Table 3 and Table 4), which showed relatively higher HLA scores. The ITScorePeP scores were between −148~−136, indicating that the predicted crystal structures were stable. All epitopes were docked inside the catalytic pocket of the receptor protein. In particular, epitope 1220-FIAGLIAIV-1228 from the spike protein possessed 2–5 stable hydrogen bonds with the HLA alleles, and epitope 4-FVSEETGTL-12 from the envelope protein possessed 4~5 stable hydrogen bonds (Table 4). Taken together, the epitopes included in our design were able to interact with the given HLA alleles, according to in silico prediction.

### 2.5. Humoral Immune Responses to SARS-CoV-2 S Protein

Based on the above immunoinformatic analysis, 4 designed peptides, namely P9, P12, P14 and P15, were chosen as the vaccine candidates for the downstream validation experiments because of their relatively higher counts of B-cell and T-cell epitopes and the higher frequencies of their epitopes’ corresponding HLA alleles (Table 3). We immunized mice by subcutaneous injection of a mixture of these synthesized peptides plus QuickAntibody (an adjuvant for stimulating B-cells). Mice injected with QuickAntibody only or PBS were considered as controls. The immunization was performed once a week and repeated four times in total.

**Figure 4:**
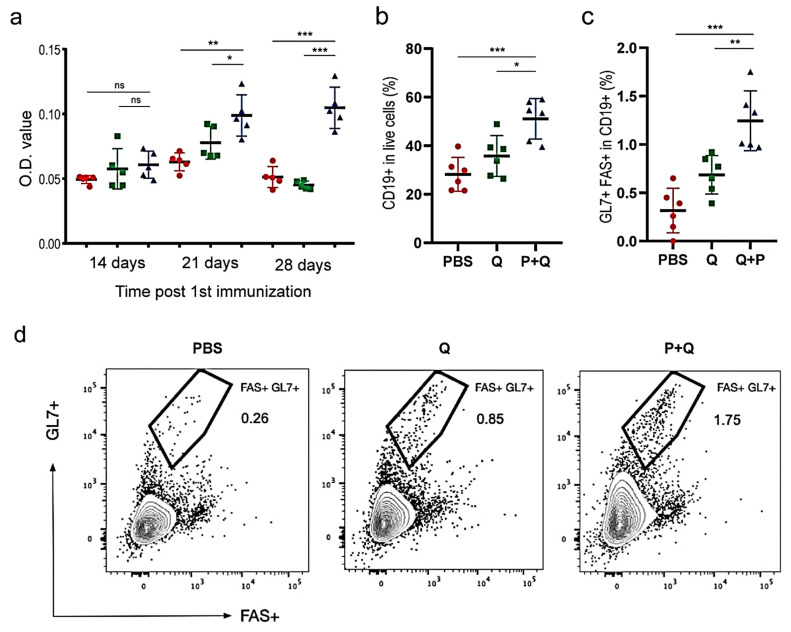
Humoral immune responses against SARS-CoV-2 S proteins. (**a**) Comparison of humoral responses among groups of mice injected with PBS (marked in red), QuickAntibody (in green) and Peptide + QuickAntibody (in blue), respectively. The level of total IgG was measured by ELISA. (**b**) The percentages of B-cells (CD19+ cells) in live cells. (**c**) The percentages of GC cells (FAS+GL7+ cells) in CD19+ cells. (**d**) Flow cytometry showing the larger percentage of FAS+/GL7+ cells in the peptide-treated mice. PBS, Q, P+Q represent mice injected with PBS, mice with QuickAntibody and mice with peptide vaccines plus QuickAntibody, respectively. * *p* < 0.05; ** *p* < 0.01; *** *p* < 0.001; ns: not significant.

To evaluate whether these peptides induced B-cells to produce S protein-specific antibodies, an ELISA assay was conducted to detect IgG in the mice sera. Fourteen days after the 1st immunization, the amount of IgG showed little difference between the peptide-treated mice and the controls (Figure 4a), suggesting that two weeks were not long enough to elicit humoral immune response. On the 21st day after the 1st immunization, however, the expression of total IgG had risen to the plateau in the peptide-treated mice and was remarkably higher than that in the control mice (*p* < 0.05; Figure 4a). Germinal centers (GCs) are the main sites for producing long-lived plasma cells and memory B-cells of high affinity. On the 28th day after the 1st immunization, inguinal lymph nodes (ILNs) were collected and then processed into single-cell suspensions for staining with the antibodies against GL7 and FAS, which were highly expressed in germinal-center B-cells and therefore considered markers. Flow cytometry showed that there were many more B-cells activated in the peptide-treated mice than in the mice injected with adjuvant only (Figure 4b); the numbers of rapidly proliferated B-cells (CD19+/FAS+/GL7+) from GC in the ILNs of the peptide-treated mice were significantly higher than those of the control groups, demonstrating increased GC induction by peptide vaccines (Figure 4c,d). In the future, a viral neutralization study is further required for demonstrating that the designed peptide vaccines can efficiently activate specific humoral immune responses to the S protein of SARS-CoV-2.

### 2.6. Cellular Immune Responses to SARS-CoV-2 S Proteins

**Figure 5:**
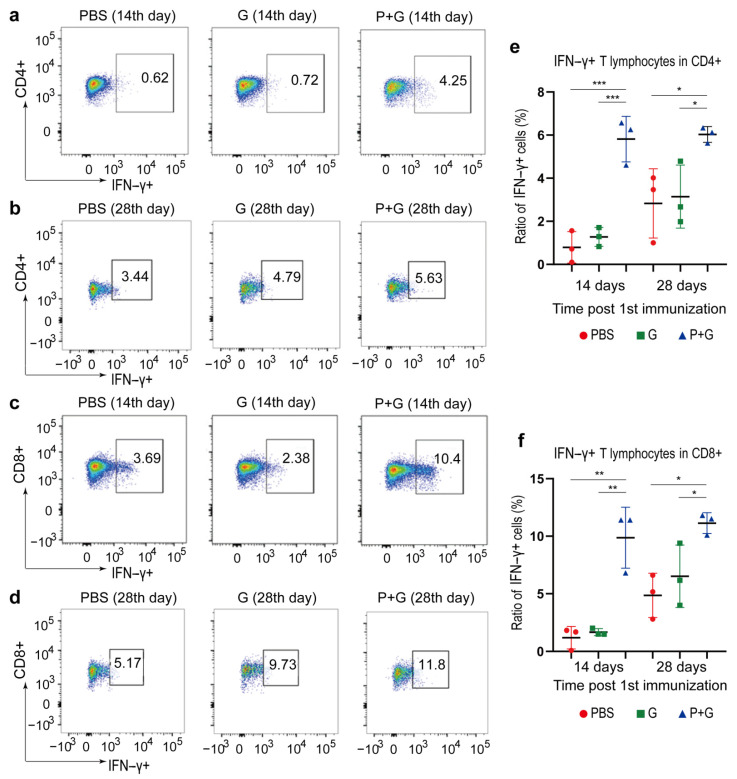
SARS-CoV-2 S protein-specific T-cell activation through peptide vaccination. (**a**,**b**) show the IFN-γ-secreting T lymphocytes in CD4+ cells on the 14th and the 28th day after the 1st immunization, respectively. (**c**,**d**) show the IFN-γ-secreting T lymphocytes in CD8+ cells on the 14th and the 28th day after the 1st immunization, respectively. (**e**,**f**) show the percentage of IFN-γ-secreting cells in CD4+ cells and CD8+ cells, respectively. PBS, G and P + G represent mice injected with PBS, GM-CSF and peptide vaccines plus GM-CSF, respectively. * *p* < 0.05; ** *p* < 0.01; *** *p* < 0.001.

In parallel, we also immunized mice with peptides plus granulocyte-macrophage colony stimulating factor (GM-CSF), an adjuvant that induced the development of monocytes, neutrophils and dendritic cells. Mice in control groups were injected with GM-CSF only or PBS. ILNs were collected on both the 14th and the 28th day after the 1st immunization. It was found that the ratios of IFN-γ-secreting cells to both CD4+ and CD8+ T-cells in the peptide-treated mice were significantly higher than those of the control groups (Figure 5), suggesting significantly stronger T-cell activation. Notably, the ratio of IFN-γ-secreting cells seemed to reach its plateau on the 14th day because no further significant increase of this ratio was observed on the 28th day. It was speculated that in the absence of the virus, the repeated T-cell stimulation led to the depletion or transferring of T-cells in the ILNs; however, the ratio of IFN-γ-secreting cells in the ILNs stayed relatively stable afterward.

**Figure 6:**
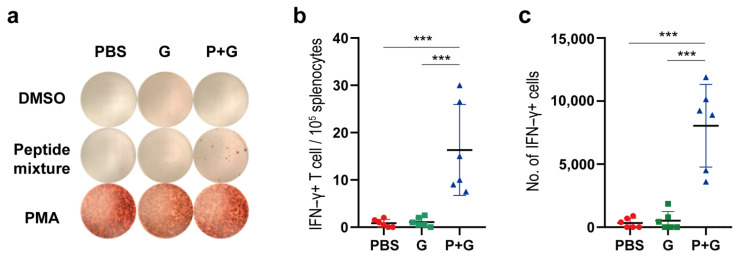
Quantification of the IFN-γ-secreting lymphocytes in mice spleens by ELISPOT. (**a**) Responses of splenocytes to DMSO (negative control), PMA (unspecific positive control) and the peptide mixture. (**b**) The number of IFN-γ-secreting cells per 100,000 splenocytes. (**c**) Total number of IFN-γ-secreting cells in the spleen. PBS, G and P+G represent mice injected with PBS, GM-CSF, and peptide vaccines plus GM-CSF, respectively. *** *p* < 0.001.

The peptide-specific lymphocytes in mice spleens were also quantified on the 28th day using IFN-γ enzyme-linked immunospot (ELISPOT) assays (Figure 6). Briefly, the splenocytes collected from PBS, GM-CSF or peptide vaccines, in combination with the GM-CSF group, were re-stimulated with a mixture of peptides, DMSO-containing culture medium (negative control) or phorbol myristate acetate (PMA) that gave unspecific positive responses, respectively. For lymphocytes collected from vaccines in combination with the GM-CSF group, both the ratio of IFN-γ-secreting lymphocytes in splenocytes and the total number of IFN-γ-secreting lymphocytes in the spleen were significantly higher in peptide-vaccinated mice compared to mice in control groups. This finding was overall consistent with the flow cytometry results of ILN cells, suggesting that the lymphocytes were activated and might recirculate to gather in the spleen after the 4-week vaccination.

## 3. Discussion

Considered one of the essential ways to halt the pandemic, the development of COVID-19 vaccines has been faced with many challenges. Currently, multiple SARS-CoV-2 variants, including the newly discovered one with double mutations in India, have been found [27]. These variants might confer immune escape through virus mutation, thereby increasing infectivity. A computation-based, immunoinformatic method that can identify viral antigens based on pathogen genome data is suitable for rapid vaccine development. In addition, vaccines developed using a multi-epitope strategy can reduce the risk of pathogens’ immune escape, as these vaccines target multiple antigens.

To date, a variety of in silico strategies based on amino acid properties such as hydrophobicity, solvent accessibility, structure, flexibility, antigenicity, etc. have been employed to obtain candidate viral epitopes from SARS-CoV-2 [28,29,30,31,32,33,34,35,36,37]. Among these predictive tools, NetCTL1.2 (http://www.cbs.dtu.dk/services/NetCTL) (accessed on 4 June 2021), a tool that integrates multi-factor algorithms for the prediction of MHC binding, proteasomal cleavage patterns and TAP transport, has been used in many studies [29,30,32,35,36]. However, a tool with more up-to-date machine learning algorithms for the prediction of SARS-CoV-2 T-cell epitopes could be of more interest, as NetCTL1.2 might be a bit outdated for lacking state-of-the-art technologies in machine learning algorithms. Schulien and Quadeer summarized that 33 T-cell epitopes associated with HLA-A*02:01, the most prevalent HLA-I allele, had demonstrated immunogenicity in vitro [38,39]. We discovered that the employment of NetMHCpan-4.0 (http://www.cbs.dtu.dk/services/NetMHCpan-4.0) (accessed on 4 June 2021) recalled 30 (90.9%) out of the 33 epitopes, whereas NetCTL1.2 only managed to recall 24 (72.7%) (Supplementary Table S3), demonstrating that prediction accuracy could be improved by using a tool with more up-to-date and better-accepted algorithms.

In recently published studies that directed attention to the prediction of SARS-CoV-2 HLA-I epitopes using different prediction tools, around half only utilized a single tool to predict T-cell epitopes. Herein, NetMHCpan-4.0 was combined with an in-house prediction platform iNeo-Pred which outperformed the former in predicting specific HLA haplotypes [40]. Previously, iNeo-Pred successfully predicted neoantigens for cancer patients; and 80% of the predicted peptides managed to elicit immune responses in an in vitro ELISpot assay [41]. Higher prediction accuracy was expected using a combinational prediction strategy. As humoral response was also important for combating SARS-CoV-2, B-cell epitopes were incorporated in our peptides. The B-cell epitopes were predicted using BepiPred2.0 (http://www.cbs.dtu.dk/services/BepiPred) (accessed on 4 June 2021), a popular tool with a balanced performance for predicting linear B-cell epitopes. Taking these together, our prediction strategy was novel, and the results of high credibility were expected.

Apart from the advantages in epitope prediction in our study, the design and selection of peptide antigens were also advantageous. Instead of grafting individual epitopes together by linkers to obtain peptide antigens [33], overlapping epitopes, consisting of both B-cell epitopes and T-cell epitopes, were incorporated in our study to construct peptides at an acceptable length for synthesis that contained sufficient epitopes. Downstream selection of immunogenic peptides based on the number of epitopes they contained as well as HLA scores ensured the immunogenicity and population coverage of the peptide vaccine. Eventually, pooled peptide antigens successfully elicited both humoral and cellular immune responses against the SARS-CoV-2 spike protein.

Until now, candidate vaccines against SARS-CoV-2 that are under development for COVID-19 include various types based on inactivated viruses, recombinant proteins, viral vectors, RNA, DNA or peptides. The involvement of optimization for cell and virus culturing conditions increased the complexity of manufacturing inactivated virus- or viral vector-based vaccines, slowing down the process for mass production [42]. Moreover, the development of inactivated virus vaccines relies heavily on extremely high manufacturing standards such as the good manufacturing practice (GMP) system to avoid medical accidents due to the failure of complete inactivation of the virus’ toxicity. Viral subunit components, especially proteins, are of highly interest for vaccine development due to their preferable properties in avoiding eliciting redundant immune responses compared to whole-pathogen approaches. Most of the recombinant protein vaccines under development now focus on the spike protein of SARS-CoV-2. These candidates might be safer to use; however, the addition of effective adjuvants to stimulate strong T-cell immune responses is often required [28]. Similarly, most viral vector vaccines under development are based on the expression of the spike protein [43]. Genetic vaccines consisting of DNA or RNA have witnessed prosperity with the development of sequencing techniques over the last few decades. However, the possibility of the integration of foreign DNA sequences into the human genome has brought uncertainty to the development of DNA vaccines, thereby limiting their application for mass immunization. To date, several mRNA vaccines have been approved by the FDA for mass immunization under Emergency Use Authorization [8,44]. However, substitutes with better safety and higher immunogenicity to provide better protection against COVID-19, are still wanted.

Peptide vaccines contain the least, as well as highly specific, components to mount an immune response, making them safe to use. However, peptide vaccines might have some disadvantages, for example, additional booster immunizations of peptide vaccines might be required to ensure sufficient immune responses against an antigen. In addition, developing a molecular adjuvant or delivery system that can boost the immune response is of vital importance for the development of peptide vaccines, as the choices of approved adjuvants or delivery systems are still limited. Compared with other platforms, peptide technologies have a relatively mature manufacturing process, as their mass production can be achieved by using solid-phase peptide synthesis. More importantly, with the help of reverse vaccinology technologies that involve epitope prediction, HLA-typing and HLA-binding affinity prediction, peptides of high immunogenicity can be designed. Thus, rapid vaccine development can be achieved during a pandemic like Covid-19 by applying peptide technologies.

In conclusion, we developed a peptide vaccine consisting of multiple B-cell and T-cell epitopes of SARS-CoV-2 using an in silico approach. GM-CSF was administered with the vaccine as an adjuvant due to ethical concerns because adjuvants such as poly-ICLC and CpG-ODNs have not been approved in China yet. Eventually, this multi-epitope-based vaccine managed to elicit antigen-specific humoral and cellular responses in a mouse model, demonstrating its potential in combating SARS-CoV-2.

## 4. Methods

### 4.1. Data Retrieval

The genome sequence of SARS-CoV-2 isolated from Wuhan-Hu-1 was retrieved from the NCBI database under the accession number MN908947. Gene and protein sequences were acquired according to the annotation. In particular, the RBD region for the spike protein was referred to as the fragment from 347 to 520 amino acid (aa) [45].

### 4.2. B-Cell Epitope Prediction

The online tools in the IEDB (Immune Epitope Database and Analysis Resource) were used for the analysis of the conserved regions of the candidate epitopes [46]. Prediction of linear B-cell epitopes was performed using Bepipred-2.0 software, with a threshold of −0.075 [47]. The antigenic sites were determined using the Kolaskar method, with a threshold of 1 [48]. The surface-accessible epitopes were predicted using the Emini tool, with a threshold of 1 [49].

### 4.3. T-Cell Epitope Prediction

The sequences of structural proteins were split into small fragments with a length of 9 aa; their binding affinity with the 34 most prevalent HLA alleles was predicted using both NetMHCpan-4.0, according to its official manual [50], and our in-house prediction software, iNeo-Pred. iNeo-Pred was trained on a large immune-peptide dataset, and achieved better performance in predicting the binding affinity of epitopes to specific HLA alleles. Only those with the top 2% binding affinity in the trained data were considered epitopes with affinity. In addition, only the epitopes predicted by both tools were selected. For all selected epitopes, corresponding HLA scores were calculated based on the frequencies of their binding HLA alleles among the Chinese population. These epitopes were further screened based on their HLA scores.

### 4.4. Vaccine Peptide Design

The vaccine peptides were designed by our in-house tool, iNeo-Design. First, the selected B-cell epitopes and their adjacent T-cell epitopes were bridged to form candidate peptides with lengths no more than 30 aa. Meanwhile, to facilitate peptide synthesis, the peptide sequences were optimized based on their physicochemical properties such as hydrophobicity and acidity. To minimize potential safety issues, peptides with toxicity potential, containing human homologous regions (full-length matches and identity >95%) or that were bioactive were excluded from the final vaccine formulation.

Apart from the peptides containing both B-cell and T-cell epitopes, iNeo-Design also utilized all predicted T-cell epitopes to generate peptides containing T-cell epitopes only. For each vaccine candidate, the epitope counts and an HLA score reflecting the population coverage were calculated. Vaccine candidates with a higher epitope count and HLA score were considered to be preferable for downstream analysis.

### 4.5. Structural Analysis

The online server SWISS-MODEL was used to predict the 3D protein structures of viral proteins and HLA molecules [51]. The online server PEP_FOLD was used to predict the structure of T-cell epitopes [52]. To display the interaction between T-cell epitopes and HLA molecules, T-cell epitope models were docked to HLA molecules using MDockPep [53]. All predicted structures or models were decorated and displayed by the open source version of the pymol program (https://github.com/schrodinger/pymol-open-source) (accessed on 4 June 2021).

### 4.6. Immuno-Stimulation of B Lymphocytes

The selected 4 peptides were synthesized using solid phase peptide synthesis by GenScript Biotech Company (Nanjing, China) and were mixed in PBS at equal mole numbers (0.03 μmol per peptide). Thirty-six female C57BL/6 mice aged 6–8 weeks old were divided into three groups (12 mice each) randomly and immunized subcutaneously with 100 μL of the following compounds: Group 1, 100 μg peptide mixture and 50 μL QuickAntibody-Mouse (Biodragen, Beijing, China); Group 2, 50 μL QuickAntibody-Mouse; and Group 3, 100 μL PBS as negative control. The immunization was performed four times in total within an interval of one week.

On the 14th, 21st and 28th days after the 1st immunization, retro-orbital blood was collected from 5 randomly selected mice in each group. The sera were analyzed for total IgG by enzyme-linked immunosorbent assay (ELISA).

ELISA was performed to determine the total serum IgG produced by mice after immunization. Briefly, recombinant 2019-nCov S-trimer protein (Novoprotein Scientific, Inc., Shanghai, China) was diluted to a final concentration of 1 µg/mL in PBS. The microtiter plates were coated by the antigen solution for 1 h at room temperature. The plates were then washed, followed by adding 200 µL of TBS-blocking buffer to block the remaining binding sites at room temperature for 1 h. Mouse serum was diluted in PBS (1:200). 100 µL diluted serum was added to each well, except for the antigen blank and assay blank wells, for incubation at room temperature for 2 h. The plates were subsequently washed, followed by adding 100 µL goat anti-mouse IgG secondary antibody (1 µg/mL in PBS) to each well, except for the assay blank wells, for incubation at room temperature for 2 h. After washing the plate, 100 µL TMB per well was added to incubate for another 1 h in the dark at room temperature. The reaction was stopped by adding 100 µL 1 M HCl per well. All plates were read on a microplate reader. The absorbance was measured at 450 nm for horseradish peroxidase (HRP)-based substrate development. On the 28th day after the 1st immunization, 6 randomly selected mice were euthanized. The ILNs were harvested and processed into single cell suspensions. The cells were stained with Zombie Aqua (BioLegend, San Diego, CA USA), APC-conjugated anti-mouse CD19 antibody (BioLegend, San Diego, CA, USA), PerCP/Cyanine5.5- conjugated anti-mouse CD95 (Fas) antibody (BioLegend, San Diego, CA, USA) and FITC-conjugated anti-mouse GL7 antibody (BioLegend, San Diego, CA, USA). The stained cells were resuspended in 500 μL PBS and subsequently processed by the Aria II flow cytometry instrument (BD, Franklin Lakes, NJ, USA).

Flow cytometry staining was performed following the steps described previously [41]. Briefly, 5 × 10^6^ splenocytes/ILNs were cultured in 24-well cell culture plates containing RPMI-1640 medium with penicillin/streptomycin (1: 100 *V*/*V* to the medium) (Gibco) and 10% FBS (Gibco). Each sample was assessed with mock (100 μL PBS containing0.5% DMSO as background control) peptide pools (each 1.5–2 μg/mL) or 10 pg/mL phorbol myristate acetate together with 1 μg/mL ionomycin (Sigma-Aldrich, St. Louis, MO, USA) (100 μL; positive control). The plates were incubated at 37 °C overnight. After incubation, 0.25 μL GolgiStop and 0.25 μL GolgiPlug in 50 μL of RPMI were added to each well, incubated at 37 °C for 8 h, and then held at 4 °C overnight. The next day, the cells were washed twice with DPBS and stained with Zombie Aqua live/dead dye for 10 min first and APC-conjugate anti-mouse CD antibody (BioLegend, San Diego, CA, USA), PerCP/Cyanine 5.5-conjugated anti-mouse CD95 (Fas) antibody (BioLegend, San Diego, CA, USA) and FITC-conjugated anti-mouse GL7 antibody (BioLegend, San Diego, CA, USA), subsequently, for 30 min. Cells were then washed twice with 2% FBS/DPBS buffer. The stained cells were resuspended in 500 μL PBS and subsequently processed by the Aria II flow cytometry instrument (BD, Franklin Lakes, NJ, USA). A minimum of 10,000 events were acquired per sample.

### 4.7. Immuno-Stimulation of T-Cells

The animal study designed for immune-stimulation of T-cells was similar to that of the B-cells, as described above. However, the compounds for injection were different. For Group 1, 100 μg peptide mixture and 10 μg granulocyte-macrophage colony stimulating factor (GM-CSF; Novoprotein, Shanghai, China) were given to each mouse; 10 μg GM-CSF was given to each mouse in Group 2 and 100 μL PBS was given to all mice in Group 3 as negative controls.

On the 14th and 28th day after the 1st immunization, 3 randomly selected mice in each group were euthanized. The relative proportions of T-cells in the splenocytes and ILN lymphocytes were analyzed by the Aria II flow cytometry instrument (BD, Franklin Lakes, NJ, USA). The IFN-γ-secreting T lymphocytes were also quantified on 6 randomly selected mice using an ELISPOT kit (Dakewe, Shenzhen, China). Splenocytes stimulated with phorbol myristate acetate (PMA) served as positive controls. Individual spots were counted under a CTL-ImmunoSpot^®^S6 FluoroSpot (Cellular Technology, Kennesaw, GA, USA).

IFN-γ ELISpot assay was performed per the kit’s instructions. Briefly, 1 × 10^5^ splenocytes/ILN lymphocytes were plated in triplicate with 100 μL pooled peptides (each 5 μg/mL) dissolved in RPMI-1640 medium, mock (RPMI-1640 medium; background control) or 1 μL 1 ug/mL ionomycin and 50 ng/mL PMA (positive control) for incubation at 37 °C overnight. The plates were then rinsed with PBS containing 0.05% Tween-20, followed by the addition of 1 µg/mL anti-mouse IFN-γ mAb for incubation at 37 °C for 1 h (Dakewe, Shenzhen, China). After rinsing with PBS containing 0.05% Tween-20, streptavidin-ALP (Dakewe, Shenzhen, China) was added for incubation for 1 h at 37 °C. The plates were then washed, followed by the addition of AEC (Dakewe, Shenzhen, China) for incubation at 37 °C for 25 min to develop immunospots. The spots were imaged and enumerated (Cellular Technology Ltd.).

### 4.8. Statistical Analysis

Comparisons were analyzed by one-way analysis of variance (ANOVA). *P* values less than 0.05 were considered significant.

## Figures and Tables

**Table 1:** B-cell epitope candidates.

Epitope	Protein	Start	End	Peptide	Emini	Kolaskar
B1	Spike	19	43	TTRTQLPPAYTNSFTRGVYYPDKVF	6.424	1.028
B2	Spike	90	99	VYFASTEKSN	1.573	1.019
B3	Spike	206	209	KHTP	2.463	1.002
B4	Spike	405	430	DEVRQIAPGQTGKIADYNYKLPDDFT	5.81	1.001
B5	Spike	494	507	SYGFQPTNGVGYQP	1.553	1.02
B6	Spike	671	688	CASYQTQTNSPRRARSVA	3.531	1.027
B7	Spike	771	782	AVEQDKNTQEVF	2.342	1.011
B8	Spike	787	799	QIYKTPPIKDFGG	1.465	1.006
B9	Spike	805	816	ILPDPSKPSKRS	4.69	1.019
B10	Spike	1052	1058	FPQSAPH	1.381	1.059
B11	Spike	1068	1091	VPAQEKNFTTAPAICHDGKAHFPR	1.063	1.03
B12	Spike	1108	1123	NFYEPQIITTDNTFVS	1.039	1.007
B13	Spike	1135	1151	NTVYDPLQPELDSFKEE	6.183	1.011
B14	Spike	1153	1172	DKYFKNHTSPDVDLGDISGI	1.399	1.007
B15	Spike	1190	1193	AKNL	1.087	1.005
B16	Spike	1203	1209	LGKYEQY	2.512	1.035
B17	Spike	1255	1265	KFDEDDSEPVL	2.654	1.003
B18	Spike	63	70	KNLNSSRV	3.471	1.002
B19	Spike	173	176	SRTL	1.504	1.011

Note: Epitopes B4 and B10 are located within the RBD region.

**Table 2:** Distribution of T-cell epitopes among three structural proteins.

Protein	Count of T-Cell Epitopes	No. of Epitopes Per Residue	Epitope Overage	HLA Type Count
Spike	378	0.297	93.01%	33
Membrane	90	0.405	96.00%	31
Envelope	31	0.413	94.14%	32

**Table 3:** Candidate vaccine peptides.

Peptide	Protein	Start	End	Vaccine Peptide	Count of T-Cell Epitopes	Count of B-Cell Epitopes	HLA Score
P1	Spike	19	46	TTRTQLPPAYTNSFTRGVYYPDKVFRSS	10	1	1.086
P2	Spike	75	99	GTKRFDNPVLPFNDGVYFASTEKSNK	6	1	1.143
P3	Spike	118	143	LIVNNATNVVIKVCEFQFCNDPFLGVKK	7	0	1.179
P4	Spike	142	170	GVYYHKNNKSWMESEFRVYSSANNCTFEY	10	0	1.664
P5	Spike	186	209	FKNLREFVFKNIDGYFKIYSKHTP	8	1	1.264
P6	Spike	258	279	WTAGAAAYYVGYLQPRTFLLKYKKKKK	10	0	1.115
P7	Spike	310	337	KGIYQTSNFRVQPTESIVRFPNITNLCP	10	0	1.012
P8 *	Spike	357	386	RISNCVADYSVLYNSASFSTFKCYGVSPTK	8	0	1.318
P9 *	Spike	405	433	DEVRQIAPGQTGKIADYNYKLPDDFTGKKK	7	1	0.928
P10 *	Spike	448	472	NYNYLYRLFRKSNLKPFERDISTEI	7	0	1.625
P11 *	Spike	478	505	TPCNGVEGFNCYFPLQSYGFQPTNGVGYKK	7	0	1.413
P12	Spike	494	523	SYGFQPTNGVGYQPYRVVVLSFELLHAPAT	10	1	1.581
P13	Spike	625	652	HADQLTPTWRVYSTGSNVFQTRAGCLIG	8	0	1.214
P14	Spike	671	699	CASYQTQTNSPRRARSVASQSIIAYTMSL	8	1	1.234
P15	Spike	771	799	AVEQDKNTQEVFAQVKQIYKTPPIKDFGGK	8	2	0.952
P16	Spike	805	833	ILPDPSKPSKRSFIEDLLFNKVTLADAGFK	7	1	1.068
P17	Spike	896	923	IPFAMQMAYRFNGIGVTQNVLYENQKLI	7	0	1.625
P18	Spike	965	991	QLSSNFGAISSVLNDILSRLDKVEAEVKKK	9	0	1.012
P19	Spike	1052	1073	FPQSAPHGVVFLHVTYVPAQEK	8	1	1.532
P20	Spike	1068	1096	VPAQEKNFTTAPAICHDGKAHFPREGVFV	4	1	0.402
P21	Spike	1095	1123	FVSNGTHWFVTQRNFYEPQIITTDNTFVSK	8	1	1.236
P22	Spike	1135	1155	NTVYDPLQPELDSFKEELDKYKKKKK	2	1	0.254
P23	Spike	1153	1181	DKYFKNHTSPDVDLGDISGINASVVNIQKK	5	1	0.322
P24	Spike	1190	1217	AKNLNESLIDLQELGKYEQYIKWPWYIWKK	6	2	0.659
P25	Spike	1216	1245	IWLGFIAGLIAIVMVTIMLCKKKKKKKKKK	5	0	1.394
P26	Spike	1236	1265	KKKKCCSCLKGCCSCGSCCKFDEDDSEPVL	4	1	0.520
P27	Envelope	4	33	FVSEETGTLIVNSVLLFLAFVVFLKKKKKK	11	0	1.133
P28	Envelope	45	70	NIVNVSLVKPSFYVYSRVKNLNSSRV	9	1	1.455
P29	Membrane	122	150	VPLHGTILTRPLLESELVIGAVILRGHLRK	9	0	1.508
P30	Membrane	173	201	SRTLSYYKLGASQRVAGDSGFAAYSRYRI	6	1	0.902

Note: Peptides labeled by asterisks (*) were located within the RBD region.

**Table 4:** Docking results for T-cell epitopes P25 and P27 with three HLA types.

Panel	Protein	Start	Epitope	HLA Type	HLA Score	ITScorePeP	Contact Residues
a	Spike	1220	FIAGLIAIV	HLA-A*02:01	0.123	−144.2	PHE-1, GLY-4, LEU-5, ILE-6, ALA-7
b	Spike	1220	FIAGLIAIV	HLA-B*46:01	0.102	−138.2	ILE-6, VAL-9
c	Spike	1220	FIAGLIAIV	HLA-C*03:04	0.100	−146.6	PHE-1, ALA-3, ILE-8, VAL-9
d	Envelope	4	FVSEETGTL	HLA-A*02:06	0.052	−147.7	PHE-1, VAL-2, SER-3, GLU-4, THR-6
e	Envelope	4	FVSEETGTL	HLA-B*46:01	0.102	−140.2	PHE-1, SER-3, GLU-4, THR-6, THR-8
f	Envelope	4	FVSEETGTL	HLA-C*07:02	0.152	−136.7	PHE-1, GLU-4, THR-8, LEU-9

Note: HLA score was calculated based on the frequencies of HLA alleles binding in the population. ITScorePeP is a metric from the MDockPep method, which was derived based on the crystal structures of protein–peptide complexes.

## Data Availability

Not applicable.

## Supplementary Materials

The following are available online at https://www.mdpi.com/article/10.3390/pathogens10060737/s1: Table S1, Information on the 61 identified B-cell epitopes; Table S2, Information on all identified T-cells; Table S3, 33 identified T-cell epitopes for HLA allele A*02:01 in vitro reported by Schulien and Quadeer and their binary prediction results by NetMHCpan-4.0 and NetCTL-1.2.
