# Supplementary material for: Multi-Epitope Vaccine Design Using an Immunoinformatic Approach for SARS-CoV-2
Source: Pathogens. 2021 Jun 11;10(6):737. doi: 10.3390/pathogens10060737 (PMC8230658; doi:10.3390/pathogens10060737)
Supplement: Supplementary file 1 [file pathogens-10-00737-s001.zip › pathogens-1190566-supplementary.pdf]

pathogens

MDPI

Table S1. Information on the 61 identified B-cell epitopes.

Protein	Start	End	Peptide	Length	Emini	Kolaskar	Selected for Downstream Analysis
Envelope	5	11	VSEETGT	7	2.26	0.97	
Envelope	63	70	KNLNSSRV	8	3.471	1.002	Yes
Membrane	1	10	MADSNGTITV	10	0.56	0.977	
Membrane	109	115	MWSFNPE	7	1.405	0.93	
Membrane	161	171	IKDLPKEITVA	11	0.934	1.05	
Membrane	173	176	SRTL	4	1.504	1.011	Yes
Membrane	181	196	LGASQRVAGDSGFAAY	16	0.361	1.034	
Membrane	204	215	YKLNTDHSSSSD	12	7.078	0.993	
Spike	19	43	TTRTQLPPAYTNSFTRGVYYPDKVF	25	6.424	1.028	Yes
Spike	70	87	VSGTNGTKRFDNPVLPFN	18	1.184	0.994	
Spike	90	99	VYFASTEKSN	10	1.573	1.019	Yes
Spike	109	113	TLDSK	5	1.724	0.993	
Spike	146	154	HKNNKSWME	9	4.547	0.9	
Spike	158	166	RVYSSANNC	9	0.766	1.052	
Spike	180	187	EGKQGNFK	8	2.738	0.918	
Spike	206	209	KHTP	4	2.463	1.002	Yes
Spike	217	222	PQGFSA	6	0.809	1.02	
Spike	248	264	YLTPGDSSSGWTAGAAA	17	0.43	0.998	
Spike	280	288	NENGTITDA	9	1.46	0.909	
Spike	293	301	LDPLSETKC	9	0.844	1.06	
Spike	311	315	GIYQT	5	0.879	1.022	
Spike	317	327	NFRVQPTESIV	11	0.817	1.046	
Spike	380	387	YGVSPTKL	8	0.95	1.073	
Spike	405	430	DEVRQIAPGQTGKIADYNYKLPDDFT	26	5.81	1.001	Yes
Spike	438	448	SNNLDSKVGGN	11	1.294	0.957	
Spike	461	485	LKPFERDISTEIYQAGSTPCNGVEG	25	0.803	1.011	
Spike	494	507	SYGFQPTNGVGYQP	14	1.553	1.02	Yes
Spike	522	534	ATVCGPKKSTNLV	13	0.38	1.069	
Spike	545	559	GLTGTGVLTESNKKF	15	0.7	0.988	
Spike	564	582	QFGRDIADTTDAVRDPQTL	19	2.476	0.995	
Spike	590	593	CSFG	4	0.25	1.097	
Spike	595	607	VSVITPGTNTSNQ	13	0.803	1.013	
Spike	615	620	VNCTEV	6	0.309	1.119	
Spike	622	644	VAIHADQLTPTWRVYSTGSNVFQ	23	0.414	1.051	
Spike	655	667	HVNNSYECDIPIG	13	0.359	1.045	
Spike	671	688	CASYQTQTNSPRRARSVA	18	3.531	1.027	Yes
Spike	699	715	LGAENSVAYSNNSIAIP	17	0.312	1.026	
Spike	731	736	MTKTSV	6	1.067	0.995	
Spike	745	749	DSTEC	5	0.97	1.01	
Spike	771	782	AVEQDKNTQEVF	12	2.342	1.011	Yes
Spike	787	799	QIYKTPPIKDFGG	13	1.465	1.006	Yes
Spike	805	816	ILPDPSKPSKRS	12	4.69	1.019	Yes
Spike	833	836	FIKQ	4	0.853	1.047	
Spike	839	844	DCLGDI	6	0.223	1.07	
Spike	882	891	ITSGWTFGAG	10	0.187	0.965	
Spike	928	946	NSAIGKIQDSLSSTASALG	19	0.311	1.016	
Spike	950	958	DVVNQNAQA	9	0.974	1.038	

Spike	968	971	SNFG	4	0.749	0.938	
Spike	986	991	KVEAEV	6	0.869	1.077	
Spike	1018	1021	IRAS	4	0.754	1.025	
Spike	1023	1027	NLAAT	5	0.632	1.013	
Spike	1039	1042	RVDF	4	0.853	1.053	
Spike	1052	1058	FPQSAPH	7	1.381	1.059	Yes
Spike	1068	1091	VPAQEKNFTTAPAICHDGKAHFPR	24	1.063	1.03	Yes
Spike	1094	1097	VFVS	4	0.259	1.217	
Spike	1108	1123	NFYEPQIITTDNTFVS	16	1.039	1.007	Yes
Spike	1135	1151	NTVYDPLQPELDSFKEE	17	6.183	1.011	Yes
Spike	1153	1172	DKYFKNHTSPDVDLGDISGI	20	1.399	1.007	Yes
Spike	1190	1193	AKNL	4	1.087	1.005	Yes
Spike	1203	1209	LGKYEQY	7	2.512	1.035	Yes
Spike	1255	1265	KFDEDDSEPVL	11	2.654	1.003	Yes

Table S2. Information on all identified T-cell epitopes.

Protein	Start	Length	Epi_AA	HLA_Count	HLA_Score	HLA_Types	Comments
Envelope	4	9	FVSEETGTL	13	0.771	HLA-A*02:06HLA-B*35:03,HLA-B*15:02,HLA-B*46:01,HLA-B*35:01,HLA-B*39:01HLA-C*07:02,HLA-C*15:02,HLA-C*03:04,HLA-C*04:01,HLA-C*03:03,HLA-C*06:02,HLA-C*12:03	
Envelope	5	9	VSEETGTLI	1	0.034	HLA-C*15:02	
Envelope	6	9	SEETGTLIV	2	0.119	HLA-B*40:01,HLA-B*40:02	
Envelope	10	9	GTLIVNSVL	1	0.034	HLA-C*15:02	
Envelope	11	9	TLIVNSVLL	2	0.208	HLA-A*02:07,HLA-A*02:01	
Envelope	12	9	LIVNSVLLF	6	0.399	HLA-A*24:02,HLA-A*26:01HLA-B*46:01,HLA-B*15:02,HLA-B*15:01,HLA-B*35:01	
Envelope	13	9	IVNSVLLFL	7	0.484	HLA-A*02:07,HLA-A*02:06,HLA-A*02:01HLA-C*15:02,HLA-C*03:03,HLA-C*03:04,HLA-C*12:03	
Envelope	15	9	NSVLLFLAF	3	0.166	HLA-B*46:01,HLA-B*15:02,HLA-B*35:01	
Envelope	16	9	SVLLFLAFV	4	0.295	HLA-A*02:03,HLA-A*02:07,HLA-A*02:06,HLA-A*02:01	
Envelope	17	9	VLLFLAFVV	3	0.260	HLA-A*02:07,HLA-A*02:06,HLA-A*02:01	
Envelope	18	9	LLFLAFVVF	3	0.097	HLA-A*32:01HLA-B*15:01,HLA-B*15:02	
Envelope	20	9	FLAFVVFLL	4	0.295	HLA-A*02:03,HLA-A*02:07,HLA-A*02:06,HLA-A*02:01	
Envelope	21	9	LAFVVFLLV	3	0.106	HLA-B*51:01,HLA-B*54:01HLA-C*12:03	
Envelope	23	9	FVVFLLVTL	7	0.440	HLA-A*02:07,HLA-A*02:06HLA-B*46:01,HLA-B*35:03HLA-C*03:04,HLA-C*03:03,HLA-C*12:03	
Envelope	25	9	VFLLVTLAI	2	0.198	HLA-A*24:02HLA-C*14:02	
Envelope	26	9	FLLVTLAIL	5	0.366	HLA-A*02:03,HLA-A*02:07,HLA-A*02:06,HLA-A*02:01HLA-C*03:03	
Envelope	29	9	VTLAILTAL	9	0.460	HLA-A*02:06,HLA-A*32:01HLA-B*46:01,HLA-B*58:01,HLA-B*57:01HLA-C*15:02,HLA-C*03:03,HLA-C*03:04,HLA-C*12:03	
Envelope	31	9	LAILTALRL	8	0.452	HLA-B*46:01,HLA-B*51:01,HLA-B*58:01,HLA-B*35:03HLA-C*15:02,HLA-C*03:03,HLA-C*03:04,HLA-C*12:03	
Envelope	34	9	LTALRLCAY	6	0.269	HLA-A*01:01HLA-B*46:01,HLA-B*15:02,HLA-B*15:01,HLA-B*35:01HLA-C*12:03	
Envelope	38	9	RLCAYCCNI	5	0.308	HLA-A*32:01,HLA-A*02:03,HLA-A*02:07,HLA-A*02:06,HLA-A*02:01	
Envelope	41	9	AYCCNIVNV	1	0.156	HLA-A*24:02	
Envelope	45	9	NIVNVSLVK	1	0.211	HLA-A*11:01	
Envelope	48	9	NVSLVKPSF	2	0.064	HLA-B*35:01,HLA-B*15:02	

Envelope	49	9	VSLVKPSFY	3	0.089	HLA-B*58:01,HLA-B*57:01HLA-C*12:03
Envelope	50	9	SLVKPSFYV	4	0.295	HLA-A*02:03,HLA-A*02:07,HLA-A*02:06,HLA-A*02:01
Envelope	51	9	LVKPSFYVY	6	0.247	HLA-A*32:01HLA-B*46:01,HLA-B*15:02,HLA-B*15:01,HLA-B*35:01HLA-C*12:03
Envelope	55	9	SFYVYSRVK	1	0.054	HLA-A*30:01
Envelope	57	9	YVYSRVKNL	10	0.614	HLA-A*02:03,HLA-A*32:01HLA-C*07:02,HLA-C*15:02,HLA-C*03:04,HLA-C*04:01,HLA-C*14:02,HLA-C*03:03,HLA-C*06:02,HLA-C*12:03
Envelope	59	9	YSRVKNLNS	1	0.054	HLA-A*30:01
Envelope	61	9	RVKNLNSSR	2	0.084	HLA-A*30:01,HLA-A*03:01
Envelope	67	9	SSRVPDLLV	2	0.088	HLA-A*30:01HLA-C*15:02
Membrane	6	9	GTITVEELK	1	0.211	HLA-A*11:01
Membrane	7	9	TITVEELKK	1	0.211	HLA-A*11:01
Membrane	15	9	KLLEQWNLV	5	0.308	HLA-A*32:01,HLA-A*02:03,HLA-A*02:07,HLA-A*02:06,HLA-A*02:01
Membrane	18	9	EQWNLVIGF	2	0.048	HLA-A*32:01HLA-B*15:02
Membrane	21	9	NLVIGFLFL	2	0.208	HLA-A*02:07,HLA-A*02:01
Membrane	22	9	LVIGFLFLT	2	0.175	HLA-A*02:06,HLA-A*02:01
Membrane	23	9	VIGFLFLTW	4	0.238	HLA-A*24:02,HLA-A*32:01HLA-B*58:01,HLA-B*57:01
Membrane	26	9	FLFLTWICL	2	0.208	HLA-A*02:07,HLA-A*02:01
Membrane	27	9	LFLTWICLL	1	0.042	HLA-C*14:02
Membrane	29	9	LTWICLLQF	3	0.082	HLA-A*32:01HLA-B*58:01,HLA-B*57:01
Membrane	31	9	WICLLQFAY	1	0.029	HLA-B*35:01
Membrane	37	9	FAYANRNRF	16	0.898	HLA-A*32:01HLA-B*15:01,HLA-B*35:03,HLA-B*58:01,HLA-B*15:02,HLA-B*46:01,HLA-B*35:01,HLA-B*51:01,HLA-B*57:01HLA-C*07:02,HLA-C*03:04,HLA-C*04:01,HLA-C*14:02,HLA-C*03:03,HLA-C*06:02,HLA-C*12:03
Membrane	38	9	AYANRNRFL	5	0.499	HLA-A*24:02HLA-C*07:02,HLA-C*14:02,HLA-C*06:02,HLA-C*04:01
Membrane	39	9	YANRNRFLY	12	0.663	HLA-A*01:01,HLA-A*32:01HLA-B*15:01,HLA-B*58:01,HLA-B*15:02,HLA-B*46:01,HLA-B*35:01,HLA-B*57:01HLA-C*07:02,HLA-C*03:03,HLA-C*06:02,HLA-C*12:03
Membrane	40	9	ANRNRFLYI	1	0.054	HLA-A*30:01
Membrane	41	9	NRNRFLYII	3	0.259	HLA-B*39:01HLA-C*07:02,HLA-C*06:02
Membrane	42	9	RNRFLYIIK	1	0.054	HLA-A*30:01

Membrane	43	9	NRFLYIIKL	4	0.301	HLA-B*39:01HLA-C*07:02,HLA-C*14:02,HLA-C*06:02
Membrane	44	9	RFLYIIKLI	2	0.168	HLA-A*24:02,HLA-A*32:01
Membrane	45	9	FLYIIKLIF	2	0.062	HLA-A*32:01HLA-B*15:01
Membrane	46	9	LYIIKLIFL	3	0.350	HLA-A*24:02HLA-C*07:02,HLA-C*14:02
Membrane	47	9	YIIKLIFLW	4	0.238	HLA-A*24:02,HLA-A*32:01HLA-B*58:01,HLA-B*57:01
Membrane	50	9	KLIFLWLLW	4	0.238	HLA-A*24:02,HLA-A*32:01HLA-B*58:01,HLA-B*57:01
Membrane	53	9	FLWLLWPVT	3	0.260	HLA-A*02:07,HLA-A*02:06,HLA-A*02:01
Membrane	54	9	LWLLWPVTL	3	0.350	HLA-A*24:02HLA-C*07:02,HLA-C*14:02
Membrane	55	9	WLLWPVTLA	4	0.295	HLA-A*02:03,HLA-A*02:07,HLA-A*02:06,HLA-A*02:01
Membrane	56	9	LLWPVTLAC	1	0.085	HLA-A*02:07
Membrane	57	9	LWPVTLACF	2	0.198	HLA-A*24:02HLA-C*14:02
Membrane	58	9	WPVTLACFV	4	0.126	HLA-B*35:01,HLA-B*51:01,HLA-B*54:01,HLA-B*35:03
Membrane	60	9	VTLACFVLA	1	0.052	HLA-A*02:06
Membrane	61	9	TLACFVLAA	4	0.295	HLA-A*02:03,HLA-A*02:07,HLA-A*02:06,HLA-A*02:01
Membrane	62	9	LACFVLAAV	6	0.329	HLA-A*02:06HLA-B*51:01,HLA-B*54:01HLA-C*03:04,HLA-C*03:03,HLA-C*12:03
Membrane	65	9	FVLAAVYRI	8	0.497	HLA-A*32:01,HLA-A*02:03,HLA-A*02:07,HLA-A*02:06,HLA-A*02:01HLA-B*51:01HLA-C*15:02,HLA-C*03:04
Membrane	67	9	LAAVYRINW	3	0.082	HLA-A*32:01HLA-B*58:01,HLA-B*57:01
Membrane	68	9	AAVYRINWI	4	0.214	HLA-C*15:02,HLA-C*03:03,HLA-C*06:02,HLA-C*12:03
Membrane	71	9	YRINWITGG	1	0.152	HLA-C*07:02
Membrane	72	9	RINWITGGI	2	0.047	HLA-A*32:01HLA-C*15:02
Membrane	76	9	ITGGIAIAM	7	0.390	HLA-B*46:01,HLA-B*15:02,HLA-B*35:01HLA-C*15:02,HLA-C*03:03,HLA-C*03:04,HLA-C*12:03
Membrane	80	9	IAIAMACLV	7	0.322	HLA-A*02:06HLA-B*58:01,HLA-B*51:01,HLA-B*54:01HLA-C*15:02,HLA-C*03:03,HLA-C*12:03
Membrane	82	9	IAMACLVGL	10	0.599	HLA-A*02:06,HLA-A*02:01HLA-B*46:01,HLA-B*58:01,HLA-B*35:03,HLA-B*35:01HLA-C*15:02,HLA-C*03:03,HLA-C*03:04,HLA-C*12:03
Membrane	83	9	AMACLVGLM	6	0.430	HLA-A*02:03,HLA-A*02:07,HLA-A*02:01HLA-B*46:01,HLA-B*15:01,HLA-B*15:02

Membrane	84	9	MACLVGLM W	3	0.082	HLA-A*32:01HLA-B*58:01,HLA-B*57:01
Membrane	87	9	LVGLMWLS Y	3	0.098	HLA-A*01:01HLA-B*35:01,HLA-B*15:02
Membrane	89	9	GLMWLSYFI	5	0.308	HLA-A*32:01,HLA-A*02:03,HLA-A*02:07,HLA-A*02:06,HLA-A*02:01
Membrane	90	9	LMWLSYFIA	3	0.260	HLA-A*02:07,HLA-A*02:06,HLA-A*02:01
Membrane	92	9	WLSYFIASF	4	0.200	HLA-A*32:01HLA-B*46:01,HLA-B*15:01,HLA-B*15:02
Membrane	93	9	LSYFIASFR	1	0.211	HLA-A*11:01
Membrane	94	9	SYFIASFRL	4	0.439	HLA-A*24:02HLA-C*07:02,HLA-C*14:02,HLA-C*06:02
Membrane	95	9	YFIASFRLF	8	0.625	HLA-A*24:02HLA-B*46:01,HLA-B*15:02,HLA-B*15:01,HLA-B*35:01HLA-C*07:02,HLA-C*14:02,HLA-C*04:01
Membrane	96	9	FIASFRLFA	4	0.240	HLA-A*02:03,HLA-A*02:06,HLA-A*02:01HLA-B*54:01
Membrane	99	9	SFRLFARTR	1	0.054	HLA-A*30:01
Membrane	101	9	RLFARTRSM	13	0.741	HLA-A*02:03,HLA-A*30:01,HLA-A*32:01HLA-B*15:01,HLA-B*15:02,HLA-B*07:02HLA-C*07:02,HLA-C*03:04,HLA-C*04:01,HLA-C*14:02,HLA-C*03:03,HLA-C*06:02,HLA-C*12:03
Membrane	102	9	LFARTRSMW	2	0.198	HLA-A*24:02HLA-C*14:02
Membrane	104	9	ARTRSMWSF	2	0.241	HLA-C*07:02,HLA-C*06:02
Membrane	105	9	RTRSMWSFN	1	0.054	HLA-A*30:01
Membrane	107	9	RSMWSFNPE	1	0.054	HLA-A*30:01
Membrane	108	9	SMWSFNPET	1	0.123	HLA-A*02:01
Membrane	110	9	WSFNPETNI	5	0.268	HLA-B*58:01,HLA-B*51:01HLA-C*15:02,HLA-C*03:04,HLA-C*12:03
Membrane	111	9	SFNPETNIL	5	0.499	HLA-A*24:02HLA-C*07:02,HLA-C*14:02,HLA-C*06:02,HLA-C*04:01
Membrane	112	9	FNPETNILL	2	0.211	HLA-C*07:02,HLA-C*04:01
Membrane	116	9	TNILLNVPL	1	0.018	HLA-B*39:01
Membrane	122	9	VPLHGTILT	1	0.030	HLA-B*54:01
Membrane	125	9	HGTILTRPL	1	0.100	HLA-C*03:04
Membrane	126	9	GTILTRPLL	1	0.034	HLA-C*15:02
Membrane	130	9	TRPLLESEL	3	0.259	HLA-B*39:01HLA-C*07:02,HLA-C*06:02
Membrane	131	9	RPLLESELV	2	0.078	HLA-B*51:01,HLA-B*07:02
Membrane	134	9	LESELVIGA	1	0.019	HLA-B*40:02
Membrane	136	9	SELVIGAVI	3	0.145	HLA-B*44:03,HLA-B*40:01,HLA-B*40:02
Membrane	138	9	LVIGAVILR	1	0.211	HLA-A*11:01
Membrane	142	9	AVILRGHLR	1	0.211	HLA-A*11:01

Membrane	144	9	ILRGHLRIA	2	0.089	HLA-A*02:03,HLA-A*30:01
Membrane	148	9	HLRIAGHHL	4	0.161	HLA-A*30:01HLA-B*15:01,HLA-B*15:02,HLA-B*07:02
Membrane	150	9	RIAGHHLGR	2	0.241	HLA-A*11:01,HLA-A*03:01
Membrane	164	9	LPKEITVAT	4	0.092	HLA-B*35:01,HLA-B*07:02,HLA-B*54:01,HLA-B*35:03
Membrane	168	9	ITVATSRTL	6	0.337	HLA-B*46:01,HLA-B*57:01HLA-C*15:02,HLA-C*03:03,HLA-C*03:04,HLA-C*12:03
Membrane	170	9	VATSRTLSY	10	0.639	HLA-A*01:01HLA-B*15:01,HLA-B*58:01,HLA-B*15:02,HLA-B*46:01,HLA-B*35:01HLA-C*07:02,HLA-C*03:03,HLA-C*06:02,HLA-C*12:03
Membrane	171	9	ATSRTLSYY	8	0.473	HLA-A*11:01,HLA-A*01:01,HLA-A*30:01HLA-B*58:01,HLA-B*15:01,HLA-B*15:02,HLA-B*57:01HLA-C*12:03
Membrane	172	9	TSRTLSYYK	3	0.296	HLA-A*11:01,HLA-A*30:01,HLA-A*03:01
Membrane	173	9	SRTLSYYKL	3	0.259	HLA-B*39:01HLA-C*07:02,HLA-C*06:02
Membrane	174	9	RTLSYYKLG	1	0.054	HLA-A*30:01
Membrane	179	9	YKLGASQRV	1	0.089	HLA-C*06:02
Membrane	188	9	AGDSGFAAY	3	0.122	HLA-A*01:01HLA-B*35:01HLA-C*04:01
Membrane	191	9	SGFAAYSRY	7	0.475	HLA-B*46:01,HLA-B*15:02,HLA-B*15:01,HLA-B*35:01HLA-C*07:02,HLA-C*06:02,HLA-C*12:03
Membrane	193	9	FAAYSRYRI	6	0.369	HLA-B*51:01HLA-C*15:02,HLA-C*03:04,HLA-C*03:03,HLA-C*06:02,HLA-C*12:03
Membrane	196	9	YSRYRIGNY	6	0.447	HLA-B*46:01,HLA-B*15:01,HLA-B*15:02HLA-C*07:02,HLA-C*06:02,HLA-C*12:03
Membrane	198	9	RYRIGNYKL	5	0.464	HLA-A*24:02,HLA-A*30:01HLA-C*07:02,HLA-C*14:02,HLA-C*04:01
Membrane	209	9	DHSSSSDNI	1	0.018	HLA-B*39:01
Membrane	211	9	SSSSDNIAL	3	0.205	HLA-C*15:02,HLA-C*03:03,HLA-C*03:04
Membrane	212	9	SSSDNIALL	6	0.465	HLA-C*07:02,HLA-C*15:02,HLA-C*03:04,HLA-C*03:03,HLA-C*06:02,HLA-C*12:03
Membrane	213	9	SSDNIALLV	3	0.087	HLA-A*01:01HLA-C*15:02,HLA-C*12:03
Spike	2	9	FVFLVLLPL	7	0.552	HLA-A*02:07,HLA-A*02:06,HLA-A*02:01HLA-B*46:01HLA-C*03:04,HLA-C*03:03,HLA-C*12:03
Spike	8	9	LPLVSSQCV	5	0.148	HLA-B*35:03,HLA-B*54:01,HLA-B*07:02,HLA-B*35:01,HLA-B*51:01
Spike	10	9	LVSSQCVNL	2	0.171	HLA-C*03:04,HLA-C*03:03
Spike	19	9	TTRTQLPPA	1	0.054	HLA-A*30:01
Spike	20	9	TRTQLPPAY	2	0.241	HLA-C*07:02,HLA-C*06:02

Spike	21	9	RTQLPPAYT	1	0.054	HLA-A*30:01
Spike	24	9	LPPAYTNSF	5	0.178	HLA-B*35:01,HLA-B*51:01,HLA-B*35:03,HLA-B*07:02HLA-C*04:01
Spike	28	9	YTNSFTRGV	6	0.329	HLA-A*02:03,HLA-A*02:06HLA-C*15:02,HLA-C*06:02,HLA-C*03:04,HLA-C*12:03
Spike	29	9	TNSFTRGVY	1	0.035	HLA-B*15:02
Spike	30	9	NSFTRGVYY	7	0.569	HLA-A*11:01,HLA-A*01:01HLA-B*35:01,HLA-B*15:02HLA-C*07:02,HLA-C*06:02,HLA-C*12:03
Spike	35	9	GVYYPDKVF	3	0.097	HLA-A*32:01HLA-B*15:01,HLA-B*15:02
Spike	37	9	YYPDKVFRS	1	0.152	HLA-C*07:02
Spike	38	9	YPDKVFRSS	2	0.052	HLA-B*54:01,HLA-B*07:02
Spike	41	9	KVFRSSVLH	3	0.296	HLA-A*11:01,HLA-A*30:01,HLA-A*03:01
Spike	42	9	VFRSSVLHS	1	0.054	HLA-A*30:01
Spike	43	9	FRSSVLHST	2	0.169	HLA-B*39:01HLA-C*07:02
Spike	46	9	SVLHSTQDL	2	0.171	HLA-C*03:04,HLA-C*03:03
Spike	47	9	VLHSTQDLF	4	0.253	HLA-A*24:02,HLA-A*32:01HLA-B*15:01,HLA-B*15:02
Spike	48	9	LHSTQDLFL	2	0.169	HLA-B*39:01HLA-C*07:02
Spike	50	9	STQDLFLPF	10	0.651	HLA-A*24:02,HLA-A*26:01,HLA-A*32:01HLA-B*35:01,HLA-B*15:01,HLA-B*15:02HLA-C*07:02,HLA-C*03:04,HLA-C*03:03,HLA-C*12:03
Spike	51	9	TQDLFLPFF	1	0.060	HLA-C*04:01
Spike	55	9	FLPFFSNVT	2	0.120	HLA-A*02:03,HLA-A*02:07
Spike	56	9	LPFFSNVTW	7	0.218	HLA-B*35:03,HLA-B*58:01,HLA-B*54:01,HLA-B*07:02,HLA-B*35:01,HLA-B*51:01,HLA-B*57:01
Spike	57	9	PFFSNVTWF	1	0.156	HLA-A*24:02
Spike	59	9	FSNVTWFHA	1	0.030	HLA-B*54:01
Spike	60	9	SNVTWFHAI	2	0.047	HLA-A*32:01HLA-C*15:02
Spike	62	9	VTWFHAIHV	4	0.228	HLA-A*02:06,HLA-A*02:01HLA-C*15:02,HLA-C*12:03
Spike	65	9	FHAIHVSGT	1	0.018	HLA-B*39:01
Spike	69	9	HVSGTNGTK	2	0.241	HLA-A*11:01,HLA-A*03:01
Spike	75	9	GTKRFDNPV	1	0.054	HLA-A*30:01
Spike	78	9	RFDNPVLPF	5	0.422	HLA-A*24:02,HLA-A*32:01HLA-C*07:02,HLA-C*14:02,HLA-C*04:01
Spike	81	9	NPVLPFNDG	1	0.030	HLA-B*54:01

Spike	83	9	VLPFNDGVY	3	0.187	HLA-B*46:01,HLA-B*15:01,HLA-B*15:02
Spike	84	9	LPFNDGVYF	8	0.274	HLA-B*35:03,HLA-B*15:02,HLA-B*54:01,HLA-B*07:02,HLA-B*35:01,HLA-B*51:01HLA-C*03:03,HLA-C*12:03
Spike	89	9	GVYFASTEK	3	0.296	HLA-A*11:01,HLA-A*30:01,HLA-A*03:01
Spike	92	9	FASTEKSNI	5	0.280	HLA-B*51:01HLA-C*15:02,HLA-C*03:03,HLA-C*03:04,HLA-C*12:03
Spike	93	9	ASTEKSNII	1	0.034	HLA-C*15:02
Spike	97	9	KSNIIRGWI	4	0.158	HLA-A*30:01HLA-B*58:01,HLA-B*57:01HLA-C*15:02
Spike	102	9	RGWIFGTTL	3	0.184	HLA-A*32:01HLA-C*03:04,HLA-C*03:03
Spike	109	9	TLDSKTQSL	4	0.314	HLA-A*02:07HLA-B*39:01HLA-C*07:02,HLA-C*04:01
Spike	113	9	KTQSLLIVN	1	0.054	HLA-A*30:01
Spike	118	9	LIVNNATNV	3	0.206	HLA-A*02:03HLA-C*03:04,HLA-C*03:03
Spike	119	9	IVNNATNVV	3	0.153	HLA-C*15:02,HLA-C*03:04,HLA-C*12:03
Spike	125	9	NVVIKVCEF	2	0.063	HLA-A*26:01HLA-B*15:02
Spike	127	9	VIKVCEFQF	1	0.013	HLA-A*32:01
Spike	132	9	EFQFCNDPF	2	0.198	HLA-A*24:02HLA-C*14:02
Spike	133	9	FQFCNDPFL	11	0.808	HLA-A*02:06,HLA-A*02:01HLA-B*40:01,HLA-B*15:01,HLA-B*15:02,HLA-B*39:01HLA-C*07:02,HLA-C*03:04,HLA-C*03:03,HLA-C*06:02,HLA-C*12:03
Spike	135	9	FCNDPFLGV	2	0.086	HLA-A*02:06HLA-C*15:02
Spike	142	9	GVYYHKNNK	3	0.296	HLA-A*11:01,HLA-A*30:01,HLA-A*03:01
Spike	144	9	YYHKNNKSW	4	0.439	HLA-A*24:02HLA-C*07:02,HLA-C*14:02,HLA-C*06:02
Spike	145	9	YHKNNKSWM	2	0.241	HLA-C*07:02,HLA-C*06:02
Spike	150	9	KSWMESEFR	1	0.211	HLA-A*11:01
Spike	151	9	SWMESEFRV	1	0.156	HLA-A*24:02
Spike	152	9	WMESEFRVY	4	0.173	HLA-B*35:01,HLA-B*15:01,HLA-B*15:02HLA-C*04:01
Spike	155	9	SEFRVYSSA	1	0.019	HLA-B*40:02
Spike	158	9	RVYSSANNC	1	0.054	HLA-A*30:01
Spike	160	9	YSSANNCTF	10	0.584	HLA-B*15:01,HLA-B*58:01,HLA-B*15:02,HLA-B*35:01,HLA-B*57:01HLA-C*07:02,HLA-C*03:04,HLA-C*04:01,HLA-C*03:03,HLA-C*12:03
Spike	162	9	SANNCTFEY	9	0.690	HLA-A*11:01,HLA-A*01:01HLA-B*15:01,HLA-B*58:01,HLA-B*15:02,HLA-B*46:01,HLA-B*35:01HLA-C*07:02,HLA-C*12:03

Spike	167	9	TFEYVSQPF	5	0.438	HLA-A*24:02HLA-B*35:01HLA-C*07:02,HLA-C*14:02,HLA-C*04:01
Spike	168	9	FEYVSQPFL	3	0.179	HLA-B*40:01,HLA-B*40:02HLA-C*04:01
Spike	169	9	EYVSQPFLM	4	0.439	HLA-A*24:02HLA-C*07:02,HLA-C*14:02,HLA-C*06:02
Spike	171	9	VSQPFLMDL	6	0.386	HLA-B*57:01HLA-C*07:02,HLA-C*03:04,HLA-C*15:02,HLA-C*03:03,HLA-C*12:03
Spike	186	9	FKNLREFVF	1	0.152	HLA-C*07:02
Spike	187	9	KNLREFVFK	2	0.266	HLA-A*11:01,HLA-A*30:01
Spike	189	9	LREFVFKNI	1	0.089	HLA-C*06:02
Spike	190	9	REFVFKNID	1	0.019	HLA-B*40:02
Spike	192	9	FVFKNIDGY	10	0.627	HLA-A*26:01HLA-B*46:01,HLA-B*15:02,HLA-B*15:01,HLA-B*35:01HLA-C*07:02,HLA-C*03:04,HLA-C*14:02,HLA-C*03:03,HLA-C*12:03
Spike	193	9	VFKNIDGYF	4	0.409	HLA-A*24:02HLA-C*07:02,HLA-C*14:02,HLA-C*04:01
Spike	195	9	KNIDGYFKI	1	0.013	HLA-A*32:01
Spike	196	9	NIDGYFKIY	3	0.098	HLA-A*01:01HLA-B*35:01,HLA-B*15:02
Spike	202	9	KIYSKHTPI	6	0.408	HLA-A*02:01,HLA-A*02:07,HLA-A*30:01,HLA-A*32:01HLA-C*15:02,HLA-C*03:04
Spike	204	9	YSKHTPINL	7	0.567	HLA-B*46:01HLA-C*07:02,HLA-C*15:02,HLA-C*03:04,HLA-C*03:03,HLA-C*06:02,HLA-C*12:03
Spike	205	9	SKHTPINLV	1	0.089	HLA-C*06:02
Spike	208	9	TPINLVRDL	3	0.089	HLA-B*51:01,HLA-B*35:03,HLA-B*07:02
Spike	212	9	LVRDLPQGF	3	0.187	HLA-B*46:01,HLA-B*15:01,HLA-B*15:02
Spike	215	9	DLPQGFSAL	1	0.028	HLA-A*26:01
Spike	216	9	LPQGFSALE	1	0.030	HLA-B*54:01
Spike	218	9	QGFSALEPL	1	0.100	HLA-C*03:04
Spike	221	9	SALEPLVDL	5	0.235	HLA-B*35:03HLA-C*15:02,HLA-C*03:03,HLA-C*03:04,HLA-C*12:03
Spike	223	9	LEPLVDLPI	2	0.119	HLA-B*40:01,HLA-B*40:02
Spike	229	9	LPIGINITR	2	0.059	HLA-B*35:01,HLA-B*54:01
Spike	235	9	ITRFQTLLA	1	0.054	HLA-A*30:01
Spike	236	9	TRFQTLLAL	4	0.318	HLA-B*39:01HLA-C*07:02,HLA-C*06:02,HLA-C*04:01
Spike	240	9	TLLALHRSY	4	0.126	HLA-A*32:01HLA-B*35:01,HLA-B*15:01,HLA-B*15:02
Spike	241	9	LLALHRSYL	7	0.525	HLA-A*02:03,HLA-A*02:07,HLA-A*02:01HLA-B*07:02HLA-C*03:04,HLA-C*03:03,HLA-C*06:02
Spike	258	9	WTAGAAAYY	10	0.565	HLA-A*01:01,HLA-A*26:01HLA-B*58:01,HLA-B*35:01,HLA-B*15:01,HLA-B*15:02HLA-C*07:02,HLA-C*03:03,HLA-C*06:02,HLA-C*12:03

Spike	259	9	TAGAAAYY V	4	0.161	HLA-A*02:06HLA-B*51:01HLA-C*15:02,HLA-C*12:03
Spike	261	9	GAAAYYVG Y	5	0.344	HLA-A*11:01HLA-B*35:01,HLA-B*15:01,HLA-B*15:02HLA-C*12:03
Spike	262	9	AAAYYVGY L	7	0.567	HLA-B*46:01HLA-C*07:02,HLA-C*15:02,HLA-C*03:04,HLA-C*03:03,HLA-C*06:02,HLA-C*12:03
Spike	265	9	YYVGYLQPR	1	0.152	HLA-C*07:02
Spike	267	9	VGYLQPRTF	4	0.362	HLA-B*46:01HLA-C*07:02,HLA-C*06:02,HLA-C*12:03
Spike	268	9	GYLQPRTFL	3	0.350	HLA-A*24:02HLA-C*07:02,HLA-C*14:02
Spike	269	9	YLQPRTFLL	13	0.996	HLA-A*32:01,HLA-A*24:02,HLA-A*02:03,HLA-A*02:07,HLA-A*02:06,HLA-A*02:01HLA-C*07:02,HLA-C*03:04,HLA-C*04:01,HLA-C*14:02,HLA-C*03:03,HLA-C*06:02,HLA-C*12:03
Spike	270	9	LQPRTFLLK	2	0.241	HLA-A*11:01,HLA-A*03:01
Spike	271	9	QPRTFLLKY	1	0.029	HLA-B*35:01
Spike	285	9	ITDAVDCAL	6	0.318	HLA-A*01:01HLA-C*15:02,HLA-C*03:04,HLA-C*04:01,HLA-C*03:03,HLA-C*12:03
Spike	288	9	AVDCALDPL	4	0.283	HLA-A*02:06HLA-C*03:04,HLA-C*03:03,HLA-C*04:01
Spike	292	9	ALDPLSETK	2	0.241	HLA-A*11:01,HLA-A*03:01
Spike	296	9	LSETKCTLK	1	0.211	HLA-A*11:01
Spike	298	9	ETKCTLKSF	2	0.063	HLA-A*26:01HLA-B*15:02
Spike	302	9	TLKSFTVEK	3	0.296	HLA-A*11:01,HLA-A*30:01,HLA-A*03:01
Spike	304	9	KSFTVEKGI	4	0.116	HLA-A*32:01HLA-B*58:01,HLA-B*57:01HLA-C*15:02
Spike	310	9	KGIYQTSNF	4	0.175	HLA-A*32:01HLA-B*46:01,HLA-B*15:01,HLA-B*57:01
Spike	311	9	GIYQTSNFR	2	0.241	HLA-A*11:01,HLA-A*03:01
Spike	312	9	IYQTSNFRV	3	0.350	HLA-A*24:02HLA-C*07:02,HLA-C*14:02
Spike	318	9	FRVQPTESI	3	0.259	HLA-B*39:01HLA-C*07:02,HLA-C*06:02
Spike	319	9	RVQPTESIV	1	0.034	HLA-C*15:02
Spike	321	9	QPTESIVRF	2	0.040	HLA-B*35:01,HLA-B*35:03
Spike	324	9	ESIVRFPNI	2	0.084	HLA-A*26:01HLA-B*51:01
Spike	326	9	IVRFPNITN	1	0.054	HLA-A*30:01
Spike	327	9	VRFPNITNL	5	0.320	HLA-B*39:01HLA-C*07:02,HLA-C*14:02,HLA-C*06:02,HLA-C*12:03
Spike	329	9	FPNITNLCP	1	0.030	HLA-B*54:01
Spike	334	9	NLCPFGEVF	2	0.085	HLA-B*15:01,HLA-B*15:02
Spike	336	9	CPFGEVFNA	2	0.059	HLA-B*35:01,HLA-B*54:01

Spike	339	9	GEVFNATRF	3	0.145	HLA-B*44:03,HLA-B*40:01,HLA-B*40:02	
Spike	340	9	EVFNATRFA	2	0.058	HLA-A*26:01HLA-B*54:01	
Spike	342	9	FNATRFASV	1	0.052	HLA-A*02:06	
Spike	343	9	NATRFASVY	4	0.185	HLA-B*46:01,HLA-B*15:02,HLA-B*35:01HLA-C*12:03	
Spike	344	9	ATRFASVYA	1	0.054	HLA-A*30:01	
Spike	345	9	TRFASVYAW	4	0.271	HLA-A*32:01HLA-B*39:01HLA-C*07:02,HLA-C*06:02	
Spike	348	9	ASVYAWNRK	2	0.266	HLA-A*11:01,HLA-A*30:01	RBD
Spike	349	9	SVYAWNRKR	2	0.241	HLA-A*11:01,HLA-A*03:01	RBD
Spike	350	9	VYAWNRKRI	3	0.350	HLA-A*24:02HLA-C*07:02,HLA-C*14:02	RBD
Spike	354	9	NRKRISNCV	1	0.089	HLA-C*06:02	RBD
Spike	355	9	RKRISNCVA	1	0.054	HLA-A*30:01	RBD
Spike	357	9	RISNCVADY	4	0.127	HLA-A*03:01,HLA-A*32:01HLA-B*15:01,HLA-B*15:02	RBD
Spike	361	9	CVADYSVLY	9	0.647	HLA-A*11:01,HLA-A*01:01,HLA-A*26:01HLA-B*35:01,HLA-B*15:01,HLA-B*15:02HLA-C*07:02,HLA-C*06:02,HLA-C*12:03	RBD
Spike	366	9	SVLYNSASF	6	0.297	HLA-A*32:01HLA-B*35:01,HLA-B*15:01,HLA-B*15:02HLA-C*03:04,HLA-C*03:03	RBD
Spike	369	9	YNSASFSTF	8	0.602	HLA-A*24:02HLA-B*46:01,HLA-B*15:02,HLA-B*15:01,HLA-B*35:01HLA-C*03:04,HLA-C*03:03,HLA-C*04:01	RBD
Spike	370	9	NSASFSTFK	3	0.296	HLA-A*11:01,HLA-A*30:01,HLA-A*03:01	RBD
Spike	372	9	ASFSTFKCY	7	0.480	HLA-A*11:01,HLA-A*01:01HLA-B*46:01,HLA-B*15:02,HLA-B*15:01,HLA-B*35:01HLA-C*12:03	RBD
Spike	374	9	FSTFKCYGV	3	0.105	HLA-A*02:06HLA-C*15:02,HLA-C*12:03	RBD
Spike	378	9	KCYGVSPTK	1	0.054	HLA-A*30:01	RBD
Spike	379	9	CYGVSPTKL	3	0.350	HLA-A*24:02HLA-C*07:02,HLA-C*14:02	RBD
Spike	392	9	FTNVYADSF	9	0.615	HLA-A*26:01HLA-B*58:01,HLA-B*46:01,HLA-B*15:01,HLA-B*15:02HLA-C*07:02,HLA-C*03:04,HLA-C*03:03,HLA-C*12:03	RBD
Spike	394	9	NVYADSFVI	6	0.274	HLA-A*02:06,HLA-A*32:01HLA-B*51:01HLA-C*15:02,HLA-C*03:04,HLA-C*12:03	RBD
Spike	399	9	SFVIRGDEV	1	0.042	HLA-C*14:02	RBD
Spike	402	9	IRGDEVRQI	2	0.241	HLA-C*07:02,HLA-C*06:02	RBD
Spike	409	9	QIAPGQTGK	2	0.241	HLA-A*11:01,HLA-A*03:01	RBD

Spike	410	9	IAPGQTGKI	1	0.056	HLA-B*51:01	RBD
Spike	411	9	APGQTGKIA	1	0.022	HLA-B*07:02	RBD
Spike	413	9	GQTGKIADY	1	0.049	HLA-B*15:01	RBD
Spike	417	9	KIADYNYKL	7	0.518	HLA-A*02:01,HLA-A*02:07,HLA-A*02:06,HLA-A*32:01HLA-C*07:02,HLA-C*15:02,HLA-C*04:01	RBD
Spike	424	9	KLPDDFTGC	2	0.137	HLA-A*02:07,HLA-A*02:06	RBD
Spike	425	9	LPDDFTGCV	3	0.098	HLA-B*51:01,HLA-B*54:01,HLA-B*35:03	RBD
Spike	433	9	VIAWNSNNL	2	0.171	HLA-C*03:04,HLA-C*03:03	RBD
Spike	444	9	KVGGNYNYL	2	0.088	HLA-A*30:01HLA-C*15:02	RBD
Spike	448	9	NYNYLYRLF	4	0.439	HLA-A*24:02HLA-C*07:02,HLA-C*14:02,HLA-C*06:02	RBD
Spike	453	9	YRLFRKSNL	4	0.301	HLA-B*39:01HLA-C*07:02,HLA-C*14:02,HLA-C*06:02	RBD
Spike	454	9	RLFRKSNLK	3	0.296	HLA-A*11:01,HLA-A*30:01,HLA-A*03:01	RBD
Spike	456	9	FRKSNLKPF	2	0.241	HLA-C*07:02,HLA-C*06:02	RBD
Spike	458	9	KSNLKPFER	1	0.211	HLA-A*11:01	RBD
Spike	462	9	KPFERDIST	2	0.052	HLA-B*54:01,HLA-B*07:02	RBD
Spike	464	9	FERDISTEI	3	0.179	HLA-B*40:01,HLA-B*40:02HLA-C*04:01	RBD
Spike	478	9	TPCNGVEGF	1	0.029	HLA-B*35:01	RBD
Spike	481	9	NGVEGFNCY	2	0.064	HLA-B*35:01,HLA-B*15:02	RBD
Spike	487	9	NCYFPLQSY	2	0.064	HLA-B*35:01,HLA-B*15:02	RBD
Spike	489	9	YFPLQSYGF	4	0.409	HLA-A*24:02HLA-C*07:02,HLA-C*14:02,HLA-C*04:01	RBD
Spike	490	9	FPLQSYGFQ	1	0.030	HLA-B*54:01	RBD
Spike	495	9	YGFQPTNGV	5	0.324	HLA-B*51:01HLA-C*03:04,HLA-C*06:02,HLA-C*04:01,HLA-C*12:03	RBD
Spike	497	9	FQPTNGVGY	3	0.187	HLA-B*46:01,HLA-B*15:01,HLA-B*15:02	RBD
Spike	503	9	VGYQPYRVV	4	0.264	HLA-B*51:01HLA-C*03:04,HLA-C*06:02,HLA-C*12:03	RBD
Spike	504	9	GYQPYRVVV	3	0.350	HLA-A*24:02HLA-C*07:02,HLA-C*14:02	RBD
Spike	505	9	YQPYRVVVL	8	0.593	HLA-A*02:07HLA-B*39:01HLA-C*07:02,HLA-C*03:04,HLA-C*04:01,HLA-C*03:03,HLA-C*06:02,HLA-C*12:03	RBD
Spike	507	9	PYRVVVLSF	1	0.156	HLA-A*24:02	RBD
Spike	509	9	RVVVLSFEL	7	0.413	HLA-A*02:07,HLA-A*02:06,HLA-A*32:01HLA-B*58:01HLA-C*15:02,HLA-C*03:03,HLA-C*03:04	RBD
Spike	511	9	VVLSFELLH	1	0.211	HLA-A*11:01	RBD

Spike	512	9	VLSFELLHA	2	0.158	HLA-A*02:03,HLA-A*02:01	RBD
Spike	515	9	FELLHAPAT	1	0.019	HLA-B*40:02	
Spike	526	9	GPKKSTNLV	1	0.022	HLA-B*07:02	
Spike	529	9	KSTNLVKNK	3	0.296	HLA-A*11:01,HLA-A*30:01,HLA-A*03:01	
Spike	533	9	LVKNKCVNF	2	0.085	HLA-B*15:01,HLA-B*15:02	
Spike	535	9	KNKCVNFNF	1	0.013	HLA-A*32:01	
Spike	550	9	GVLTESNKK	2	0.241	HLA-A*11:01,HLA-A*03:01	
Spike	554	9	ESNKKFLPF	3	0.092	HLA-A*26:01HLA-B*35:01,HLA-B*15:02	
Spike	560	9	LPFQQFGRD	1	0.030	HLA-B*54:01	
Spike	568	9	DIADTTDAV	1	0.028	HLA-A*26:01	
Spike	576	9	VRDPQTLEI	4	0.318	HLA-B*39:01HLA-C*07:02,HLA-C*06:02,HLA-C*04:01	
Spike	582	9	LEILDITPC	1	0.019	HLA-B*40:02	
Spike	584	9	ILDITPCSF	5	0.229	HLA-A*01:01HLA-B*35:01,HLA-B*15:02HLA-C*03:03,HLA-C*04:01	
Spike	587	9	ITPCSFGGV	2	0.137	HLA-A*02:07,HLA-A*02:06	
Spike	590	9	CSFGGVSVI	5	0.268	HLA-B*58:01,HLA-B*51:01HLA-C*15:02,HLA-C*03:04,HLA-C*12:03	
Spike	603	9	NTSNQVAVL	3	0.190	HLA-C*03:04,HLA-C*03:03,HLA-C*12:03	
Spike	604	9	TSNQVAVLY	8	0.501	HLA-A*11:01,HLA-A*01:01HLA-B*58:01,HLA-B*15:02,HLA-B*46:01,HLA-B*35:01,HLA-B*57:01HLA-C*12:03	
Spike	612	9	YQDVNCTEV	6	0.409	HLA-A*02:07,HLA-A*02:06,HLA-A*02:01HLA-B*39:01HLA-C*03:03,HLA-C*04:01	
Spike	624	9	IHADQLTPT	1	0.018	HLA-B*39:01	
Spike	625	9	HADQLTPTW	4	0.158	HLA-B*58:01,HLA-B*35:01,HLA-B*57:01HLA-C*04:01	
Spike	628	9	QLTPTWRVY	3	0.113	HLA-B*35:01,HLA-B*15:01,HLA-B*15:02	
Spike	630	9	TPTWRVYST	2	0.052	HLA-B*54:01,HLA-B*07:02	
Spike	634	9	RVYSTGSNV	8	0.415	HLA-A*02:03,HLA-A*30:01,HLA-A*32:01HLA-C*15:02,HLA-C*03:04,HLA-C*03:03,HLA-C*06:02,HLA-C*12:03	
Spike	635	9	VYSTGSNVF	3	0.350	HLA-A*24:02HLA-C*07:02,HLA-C*14:02	
Spike	642	9	VFQTRAGCL	2	0.102	HLA-C*14:02,HLA-C*04:01	
Spike	643	9	FQTRAGCLI	3	0.169	HLA-A*02:06HLA-B*39:01HLA-C*03:04	
Spike	644	9	QTRAGCLIG	1	0.054	HLA-A*30:01	

Spike	652	9	GAEHVNNS Y	2	0.063	HLA-A*01:01HLA-B*35:01
Spike	654	9	EHVNNSYEC	1	0.018	HLA-B*39:01
Spike	658	9	NSYECDIPI	3	0.190	HLA-B*51:01HLA-C*15:02,HLA-C*03:04
Spike	660	9	YECDIPIGA	1	0.019	HLA-B*40:02
Spike	664	9	IPIGAGICA	4	0.092	HLA-B*35:01,HLA-B*07:02,HLA-B*54:01,HLA-B*35:03
Spike	666	9	IGAGICASY	5	0.235	HLA-B*46:01,HLA-B*15:02,HLA-B*15:01,HLA-B*35:01HLA-C*12:03
Spike	679	9	NSPRRARSV	1	0.089	HLA-C*06:02
Spike	680	9	SPRRARSVA	2	0.052	HLA-B*54:01,HLA-B*07:02
Spike	683	9	RARSVASQS	1	0.054	HLA-A*30:01
Spike	684	9	ARSVASQSI	3	0.259	HLA-B*39:01HLA-C*07:02,HLA-C*06:02
Spike	685	9	RSVASQSII	4	0.158	HLA-A*30:01HLA-B*58:01,HLA-B*57:01HLA-C*15:02
Spike	687	9	VASQSIIAY	8	0.399	HLA-A*01:01HLA-B*15:01,HLA-B*58:01,HLA-B*15:02,HLA-B*46:01,HLA-B*35:01HLA-C*03:03,HLA-C*12:03
Spike	689	9	SQSIIAYTM	4	0.174	HLA-B*15:01,HLA-B*39:01,HLA-B*15:02HLA-C*03:03
Spike	691	9	SIIAYTMSL	15	0.909	HLA-A*02:01,HLA-A*02:07,HLA-A*02:06,HLA-A*32:01HLA-B*15:01,HLA-B*35:03,HLA-B*15:02,HLA-B*46:01,HLA-B*07:02,HLA-B*39:01HLA-C*07:02,HLA-C*03:04,HLA-C*15:02,HLA-C*14:02,HLA-C*03:03
Spike	693	9	IAYTMSLGA	3	0.149	HLA-B*54:01HLA-C*03:04,HLA-C*12:03
Spike	697	9	MSLGAENSV	5	0.261	HLA-A*02:06HLA-B*51:01HLA-C*15:02,HLA-C*03:04,HLA-C*12:03
Spike	699	9	LGAENSVAY	6	0.306	HLA-B*46:01,HLA-B*15:02,HLA-B*15:01,HLA-B*35:01HLA-C*03:03,HLA-C*12:03
Spike	704	9	SVAYSNNSI	4	0.140	HLA-A*32:01HLA-B*07:02HLA-C*15:02,HLA-C*03:03
Spike	705	9	VAYSNNSIA	3	0.149	HLA-B*54:01HLA-C*03:04,HLA-C*12:03
Spike	706	9	AYSNNSIAI	3	0.350	HLA-A*24:02HLA-C*07:02,HLA-C*14:02
Spike	710	9	NSIAIPTNF	8	0.426	HLA-B*58:01,HLA-B*15:02,HLA-B*46:01,HLA-B*35:01,HLA-B*57:01HLA-C*03:04,HLA-C*03:03,HLA-C*12:03
Spike	712	9	IAIPTNFTI	12	0.556	HLA-A*02:06,HLA-A*32:01HLA-B*35:03,HLA-B*58:01,HLA-B*46:01,HLA-B*35:01,HLA-B*51:01,HLA-B*57:01HLA-C*15:02,HLA-C*03:03,HLA-C*03:04,HLA-C*12:03
Spike	714	9	IPTNFTISV	5	0.148	HLA-B*35:03,HLA-B*54:01,HLA-B*07:02,HLA-B*35:01,HLA-B*51:01
Spike	718	9	FTISVTTEI	11	0.737	HLA-A*02:01,HLA-A*02:06,HLA-A*26:01HLA-B*46:01,HLA-B*58:01HLA-C*15:02,HLA-C*03:04,HLA-C*04:01,HLA-C*03:03,HLA-C*06:02,HLA-C*12:03

Spike	721	9	SVTTEILPV	6	0.348	HLA-A*02:03,HLA-A*02:07,HLA-A*02:06,HLA-A*02:01HLA-C*15:02,HLA-C*12:03
Spike	725	9	EILPVSMTK	1	0.211	HLA-A*11:01
Spike	727	9	LPVSMTKTS	1	0.030	HLA-B*54:01
Spike	732	9	TKTSVDCTM	1	0.018	HLA-B*39:01
Spike	733	9	KTSVDCTMY	3	0.104	HLA-A*01:01HLA-B*58:01,HLA-B*57:01
Spike	734	9	TSVDCTMYI	5	0.253	HLA-A*02:06HLA-B*58:01HLA-C*15:02,HLA-C*06:02,HLA-C*12:03
Spike	746	9	STECSNLLL	2	0.068	HLA-A*01:01HLA-C*15:02
Spike	751	9	NLLLQYGSF	1	0.035	HLA-B*15:02
Spike	755	9	QYGSFCTQL	3	0.350	HLA-A*24:02HLA-C*07:02,HLA-C*14:02
Spike	757	9	GSFCTQLNR	1	0.211	HLA-A*11:01
Spike	759	9	FCTQLNRAL	3	0.182	HLA-B*35:03HLA-C*03:04,HLA-C*03:03
Spike	762	9	QLNRALTGI	1	0.035	HLA-A*02:03
Spike	764	9	NRALTGIAV	3	0.259	HLA-B*39:01HLA-C*07:02,HLA-C*06:02
Spike	773	9	EQDKNTQEV	1	0.018	HLA-B*39:01
Spike	777	9	NTQEVFAQV	2	0.086	HLA-A*02:06HLA-C*15:02
Spike	780	9	EVFAQVKQI	2	0.084	HLA-A*26:01HLA-B*51:01
Spike	781	9	VFAQVKQIY	2	0.194	HLA-C*07:02,HLA-C*14:02
Spike	782	9	FAQVKQIYK	3	0.296	HLA-A*11:01,HLA-A*30:01,HLA-A*03:01
Spike	786	9	KQIYKTPPI	4	0.237	HLA-A*02:01,HLA-A*02:06,HLA-A*32:01HLA-B*15:01
Spike	787	9	QIYKTPPIK	3	0.296	HLA-A*11:01,HLA-A*30:01,HLA-A*03:01
Spike	789	9	YKTPPIKDF	2	0.241	HLA-C*07:02,HLA-C*06:02
Spike	794	9	IKDFGGFNF	1	0.060	HLA-C*04:01
Spike	797	9	FGGFNFSQI	2	0.156	HLA-B*51:01HLA-C*03:04
Spike	798	9	GGFNFSQIL	1	0.019	HLA-C*12:03
Spike	803	9	SQILPDPSK	1	0.211	HLA-A*11:01
Spike	810	9	SKPSKRSFI	1	0.089	HLA-C*06:02
Spike	814	9	KRSFIEDLL	3	0.259	HLA-B*39:01HLA-C*07:02,HLA-C*06:02
Spike	815	9	RSFIEDLLF	6	0.185	HLA-A*32:01HLA-B*58:01,HLA-B*15:01,HLA-B*57:01HLA-C*15:02,HLA-C*12:03
Spike	817	9	FIEDLLFNK	1	0.211	HLA-A*11:01
Spike	818	9	IEDLLFNKV	2	0.119	HLA-B*40:01,HLA-B*40:02

Spike	821	9	LLFNKVTLA	4	0.295	HLA-A*02:03,HLA-A*02:07,HLA-A*02:06,HLA-A*02:01
Spike	825	9	KVTLADAGF	2	0.024	HLA-A*32:01HLA-B*57:01
Spike	826	9	VTLADAGFI	1	0.034	HLA-C*15:02
Spike	827	9	TLADAGFIK	2	0.241	HLA-A*11:01,HLA-A*03:01
Spike	829	9	ADAGFIKQY	1	0.026	HLA-B*44:03
Spike	833	9	FIKQYGDCL	2	0.171	HLA-C*03:04,HLA-C*03:03
Spike	852	9	AQKFNGLT V	2	0.104	HLA-A*30:01HLA-B*15:01
Spike	853	9	QKFNGLTVL	4	0.278	HLA-B*39:01HLA-C*07:02,HLA-C*06:02,HLA-C*12:03
Spike	857	9	GLTVLPPLL	2	0.208	HLA-A*02:07,HLA-A*02:01
Spike	861	9	LPPLLTDEM	4	0.118	HLA-B*35:01,HLA-B*51:01,HLA-B*35:03,HLA-B*07:02
Spike	865	9	LTDEMIAQY	5	0.177	HLA-A*01:01HLA-B*35:01,HLA-B*15:02HLA-C*04:01,HLA-C*12:03
Spike	868	9	EMIAQYTSA	1	0.028	HLA-A*26:01
Spike	869	9	MIAQYTSAL	17	0.941	HLA-A*32:01,HLA-A*02:03,HLA-A*02:07,HLA-A*02:06,HLA-A*02:01HLA-B*15:01,HLA-B*35:03,HLA-B*15:02,HLA-B*46:01,HLA-B*07:02,HLA-B*39:01HLA-C*15:02,HLA-C*03:04,HLA-C*04:01,HLA-C*14:02,HLA-C*03:03,HLA-C*06:02
Spike	870	9	IAQYTSALL	7	0.373	HLA-B*46:01,HLA-B*35:03,HLA-B*15:02HLA-C*15:02,HLA-C*03:03,HLA-C*03:04,HLA-C*12:03
Spike	871	9	AQYTSALLA	1	0.052	HLA-A*02:06
Spike	874	9	TSALLAGTI	2	0.090	HLA-B*51:01HLA-C*15:02
Spike	878	9	LAGTITSGW	2	0.070	HLA-B*58:01,HLA-B*57:01
Spike	880	9	GTITSGWTF	6	0.323	HLA-A*24:02,HLA-A*32:01HLA-B*58:01,HLA-B*15:01,HLA-B*15:02,HLA-B*57:01
Spike	886	9	WTFGAGAA L	12	0.531	HLA-A*02:06HLA-B*15:01,HLA-B*15:02,HLA-B*07:02,HLA-B*35:01,HLA-B*39:01HLA-C*15:02,HLA-C*03:04,HLA-C*04:01,HLA-C*14:02,HLA-C*03:03,HLA-C*12:03
Spike	888	9	FGAGAALQI	5	0.336	HLA-B*51:01HLA-C*03:04,HLA-C*03:03,HLA-C*06:02,HLA-C*12:03
Spike	890	9	AGAALQIPF	2	0.151	HLA-B*46:01,HLA-B*15:01
Spike	892	9	AALQIPFAM	9	0.460	HLA-B*35:03,HLA-B*58:01,HLA-B*15:02,HLA-B*46:01,HLA-B*35:01HLA-C*15:02,HLA-C*03:04,HLA-C*03:03,HLA-C*12:03
Spike	894	9	LQIPFAMQM	11	0.721	HLA-A*02:06HLA-B*46:01,HLA-B*15:01,HLA-B*39:01,HLA-B*15:02HLA-C*07:02,HLA-C*15:02,HLA-C*03:04,HLA-C*03:03,HLA-C*06:02,HLA-C*12:03

Spike	896	9	IPFAMQMAY	6	0.233	HLA-B*35:03,HLA-B*15:02,HLA-B*54:01,HLA-B*35:01,HLA-B*51:01HLA-C*03:03
Spike	898	9	FAMQMAYRF	16	0.902	HLA-A*24:02,HLA-A*32:01HLA-B*15:01,HLA-B*35:03,HLA-B*58:01,HLA-B*15:02,HLA-B*46:01,HLA-B*35:01,HLA-B*51:01,HLA-B*57:01HLA-C*03:04,HLA-C*04:01,HLA-C*14:02,HLA-C*03:03,HLA-C*06:02,HLA-C*12:03
Spike	901	9	QMAYRFNGI	2	0.048	HLA-A*02:03,HLA-A*32:01
Spike	903	9	AYRFNGIGV	2	0.097	HLA-A*30:01HLA-C*14:02
Spike	904	9	YRFNGIGVT	2	0.169	HLA-B*39:01HLA-C*07:02
Spike	909	9	IGVTQNVLY	1	0.029	HLA-B*35:01
Spike	915	9	VLYENQKLI	3	0.209	HLA-A*02:03,HLA-A*02:07HLA-C*06:02
Spike	919	9	NQKLIANQF	1	0.035	HLA-B*15:02
Spike	922	9	LIANQFNSA	3	0.117	HLA-A*02:03,HLA-A*02:06HLA-B*54:01
Spike	923	9	IANQFNSAI	8	0.429	HLA-B*46:01,HLA-B*51:01,HLA-B*35:03,HLA-B*15:02HLA-C*15:02,HLA-C*03:03,HLA-C*03:04,HLA-C*12:03
Spike	925	9	NQFNSAIGK	2	0.241	HLA-A*11:01,HLA-A*03:01
Spike	937	9	SLSSTASAL	6	0.312	HLA-A*02:03HLA-B*15:01,HLA-B*15:02,HLA-B*07:02HLA-C*03:04,HLA-C*03:03
Spike	939	9	SSTASALGK	3	0.296	HLA-A*11:01,HLA-A*30:01,HLA-A*03:01
Spike	940	9	STASALGKL	4	0.224	HLA-C*15:02,HLA-C*03:03,HLA-C*03:04,HLA-C*12:03
Spike	943	9	SALGKLQDV	5	0.276	HLA-A*02:06HLA-C*15:02,HLA-C*03:03,HLA-C*03:04,HLA-C*12:03
Spike	950	9	DVVNQNAQA	1	0.028	HLA-A*26:01
Spike	951	9	VVNQNAQAL	5	0.237	HLA-B*35:03,HLA-B*07:02HLA-C*15:02,HLA-C*03:04,HLA-C*03:03
Spike	955	9	NAQALNTLV	1	0.056	HLA-B*51:01
Spike	956	9	AQALNTLVK	2	0.241	HLA-A*11:01,HLA-A*03:01
Spike	958	9	ALNTLVKQL	1	0.035	HLA-A*02:03
Spike	962	9	LVKQLSSNF	4	0.215	HLA-B*46:01,HLA-B*15:02,HLA-B*15:01,HLA-B*35:01
Spike	964	9	KQLSSNFGA	1	0.052	HLA-A*02:06
Spike	965	9	QLSSNFGAI	1	0.035	HLA-A*02:03
Spike	968	9	SNFGAISSV	2	0.053	HLA-C*15:02,HLA-C*12:03

Spike	969	9	NFGAISSVL	3	0.258	HLA-A*24:02HLA-C*14:02,HLA-C*04:01
Spike	972	9	AISSVLNDI	1	0.035	HLA-A*02:03
Spike	973	9	ISSVLNDIL	5	0.274	HLA-B*58:01,HLA-B*57:01HLA-C*15:02,HLA-C*03:03,HLA-C*03:04
Spike	975	9	SVLNDILSR	1	0.211	HLA-A*11:01
Spike	976	9	VLNDILSRL	6	0.457	HLA-A*02:03,HLA-A*02:07,HLA-A*02:06,HLA-A*02:01HLA-B*46:01HLA-C*04:01
Spike	981	9	LSRLDKVEA	1	0.054	HLA-A*30:01
Spike	983	9	RLDKVEAEV	3	0.268	HLA-A*02:07,HLA-A*02:01HLA-C*04:01
Spike	989	9	AEVQIDRLI	3	0.145	HLA-B*44:03,HLA-B*40:01,HLA-B*40:02
Spike	996	9	LITGRLQSL	3	0.190	HLA-C*03:04,HLA-C*03:03,HLA-C*12:03
Spike	999	9	GRLQSLQTY	2	0.241	HLA-C*07:02,HLA-C*06:02
Spike	1000	9	RLQSLQTYV	4	0.295	HLA-A*02:03,HLA-A*02:07,HLA-A*02:06,HLA-A*02:01
Spike	1004	9	LQTYVTQQL	5	0.337	HLA-B*40:01,HLA-B*15:01,HLA-B*39:01HLA-C*03:04,HLA-C*03:03
Spike	1005	9	QTYVTQQLI	6	0.268	HLA-B*58:01,HLA-B*51:01,HLA-B*57:01HLA-C*15:02,HLA-C*06:02,HLA-C*12:03
Spike	1012	9	LIRAAEIRA	1	0.054	HLA-A*30:01
Spike	1014	9	RAAEIRASA	3	0.156	HLA-A*30:01HLA-B*54:01HLA-C*03:03
Spike	1016	9	AEIRASANL	3	0.145	HLA-B*44:03,HLA-B*40:01,HLA-B*40:02
Spike	1018	9	IRASANLAA	1	0.018	HLA-B*39:01
Spike	1020	9	ASANLAATK	3	0.296	HLA-A*11:01,HLA-A*30:01,HLA-A*03:01
Spike	1021	9	SANLAATKM	8	0.401	HLA-B*46:01,HLA-B*15:02,HLA-B*35:03,HLA-B*35:01HLA-C*15:02,HLA-C*03:03,HLA-C*03:04,HLA-C*12:03
Spike	1026	9	ATKMSECVL	1	0.054	HLA-A*30:01
Spike	1048	9	HLMSFPQSA	2	0.158	HLA-A*02:03,HLA-A*02:01
Spike	1050	9	MSFPQSAPH	2	0.131	HLA-B*46:01,HLA-B*35:01
Spike	1052	9	FPQSAPHGV	6	0.208	HLA-B*35:03,HLA-B*54:01,HLA-B*07:02,HLA-B*35:01,HLA-B*51:01HLA-C*04:01
Spike	1054	9	QSAPHGVVF	11	0.639	HLA-A*32:01HLA-B*15:01,HLA-B*58:01,HLA-B*15:02,HLA-B*46:01,HLA-B*35:01,HLA-B*57:01HLA-C*07:02,HLA-C*03:04,HLA-C*03:03,HLA-C*12:03
Spike	1055	9	SAPHGVVFL	5	0.401	HLA-C*07:02,HLA-C*03:04,HLA-C*04:01,HLA-C*03:03,HLA-C*12:03
Spike	1056	9	APHGVVFLH	1	0.029	HLA-B*35:01
Spike	1059	9	GVVFLHVTY	3	0.097	HLA-A*32:01HLA-B*15:01,HLA-B*15:02

Spike	1060	9	VVFLHVTYV	8	0.468	HLA-A*02:03,HLA-A*02:07,HLA-A*02:06,HLA-A*02:01HLA-B*54:01HLA-C*15:02,HLA-C*06:02,HLA-C*12:03
Spike	1062	9	FLHVTYVPA	5	0.325	HLA-A*02:03,HLA-A*02:07,HLA-A*02:06,HLA-A*02:01HLA-B*54:01
Spike	1065	9	VTYVPAQEK	3	0.296	HLA-A*11:01,HLA-A*30:01,HLA-A*03:01
Spike	1073	9	KNFTTAPAI	1	0.013	HLA-A*32:01
Spike	1086	9	KAHFPREGV	2	0.088	HLA-A*30:01HLA-C*15:02
Spike	1087	9	AHFPREGVF	3	0.259	HLA-B*39:01HLA-C*07:02,HLA-C*06:02
Spike	1088	9	HFPREGVFV	1	0.042	HLA-C*14:02
Spike	1089	9	FPREGVFVS	2	0.059	HLA-B*35:01,HLA-B*54:01
Spike	1094	9	VFVSNGTHW	1	0.156	HLA-A*24:02
Spike	1095	9	FVSNGTHWF	12	0.890	HLA-A*24:02,HLA-A*26:01HLA-B*46:01,HLA-B*35:01,HLA-B*15:01,HLA-B*15:02HLA-C*07:02,HLA-C*03:04,HLA-C*04:01,HLA-C*03:03,HLA-C*06:02,HLA-C*12:03
Spike	1096	9	VSNGTHWFV	2	0.092	HLA-B*58:01HLA-C*15:02
Spike	1099	9	GTHWFVTQR	1	0.211	HLA-A*11:01
Spike	1101	9	HWFVTQRNF	3	0.350	HLA-A*24:02HLA-C*07:02,HLA-C*14:02
Spike	1102	9	WFVTQRNFY	1	0.042	HLA-C*14:02
Spike	1106	9	QRNFYEPQI	1	0.089	HLA-C*06:02
Spike	1109	9	FYEPQIITT	1	0.152	HLA-C*07:02
Spike	1113	9	QIITTDNTF	5	0.243	HLA-A*26:01HLA-B*46:01,HLA-B*15:02,HLA-B*15:01,HLA-B*35:01
Spike	1120	9	TFVSGNCDV	1	0.042	HLA-C*14:02
Spike	1121	9	FVSGNCDVV	8	0.519	HLA-A*02:03,HLA-A*02:07,HLA-A*02:06,HLA-A*02:01HLA-C*15:02,HLA-C*03:03,HLA-C*03:04,HLA-C*12:03
Spike	1130	9	IGIVNNTVY	3	0.166	HLA-B*46:01,HLA-B*15:02,HLA-B*35:01
Spike	1137	9	VYDPLQPEL	3	0.254	HLA-C*07:02,HLA-C*14:02,HLA-C*04:01
Spike	1147	9	SFKEELDKY	1	0.042	HLA-C*14:02
Spike	1158	9	NHTSPDVDL	1	0.018	HLA-B*39:01
Spike	1161	9	SPDVDLGDI	1	0.011	HLA-B*35:03
Spike	1169	9	ISGINASVV	1	0.034	HLA-C*15:02

Spike	1171	9	GINASVVNI	2	0.048	HLA-A*02:03,HLA-A*32:01
Spike	1173	9	NASVVNIQK	1	0.211	HLA-A*11:01
Spike	1175	9	SVVNIQKEI	1	0.034	HLA-C*15:02
Spike	1181	9	KEIDRLNEV	4	0.197	HLA-A*02:06HLA-B*44:03,HLA-B*40:01,HLA-B*40:02
Spike	1185	9	RLNEVAKNL	2	0.048	HLA-A*02:03,HLA-A*32:01
Spike	1188	9	EVAKNLNES	1	0.028	HLA-A*26:01
Spike	1189	9	VAKNLNESL	2	0.171	HLA-C*03:04,HLA-C*03:03
Spike	1192	9	NLNESLIDL	2	0.158	HLA-A*02:03,HLA-A*02:01
Spike	1201	9	QELGKYEQY	1	0.026	HLA-B*44:03
Spike	1206	9	YEQYIKWPW	2	0.046	HLA-B*44:03,HLA-B*40:02
Spike	1207	9	EQYIKWPWY	1	0.035	HLA-B*15:02
Spike	1208	9	QYIKWPWYI	3	0.396	HLA-A*24:02HLA-C*07:02,HLA-C*06:02
Spike	1209	9	YIKWPWYIW	3	0.179	HLA-A*24:02,HLA-A*32:01HLA-B*57:01
Spike	1212	9	WPWYIWLGF	4	0.092	HLA-B*35:01,HLA-B*54:01,HLA-B*35:03,HLA-B*07:02
Spike	1213	9	PWYIWLGFI	1	0.156	HLA-A*24:02
Spike	1216	9	IWLGFIAGL	2	0.307	HLA-A*24:02HLA-C*07:02
Spike	1217	9	WLGFIAGLI	1	0.035	HLA-A*02:03
Spike	1218	9	LGFIAGLIA	1	0.030	HLA-B*54:01
Spike	1219	9	GFIAGLIAI	1	0.042	HLA-C*14:02
Spike	1220	9	FIAGLIAIV	8	0.550	HLA-A*02:03,HLA-A*02:07,HLA-A*02:06,HLA-A*02:01HLA-B*46:01HLA-C*15:02,HLA-C*03:04,HLA-C*12:03
Spike	1221	9	IAGLIAIVM	4	0.210	HLA-B*35:01,HLA-B*35:03HLA-C*03:04,HLA-C*03:03
Spike	1223	9	GLIAIVMVT	1	0.123	HLA-A*02:01
Spike	1224	9	LIAIVMVTI	1	0.013	HLA-A*32:01
Spike	1225	9	IAIVMVTIM	8	0.426	HLA-B*35:03,HLA-B*58:01,HLA-B*15:02,HLA-B*46:01,HLA-B*35:01HLA-C*03:04,HLA-C*03:03,HLA-C*12:03
Spike	1229	9	MVTIMLCCM	2	0.047	HLA-B*35:03,HLA-B*15:02
Spike	1236	9	CMTSCCSCL	1	0.085	HLA-A*02:07
Spike	1237	9	MTSCCSCLK	2	0.266	HLA-A*11:01,HLA-A*30:01

Spike	1248	9	CSCGSCCKF	2	0.070	HLA-B*58:01,HLA-B*57:01
Spike	1257	9	DEDDSEPVL	1	0.100	HLA-B*40:01
Spike	1262	9	EPVLKGVKL	2	0.033	HLA-B*35:03,HLA-B*07:02
Spike	1264	9	VLKGVKLH Y	2	0.085	HLA-B*15:01,HLA-B*15:02

Table S3. 33 identified T-cell epitopes for HLA allele A*02:01 in vitro reported by Schulien and Quadeer and their binary prediction results by NetMHCpan-4.0 and NetCTL-1.2.

Epitope	Length	NetMHCpan-4.0 *	NetCTL-1.2 **
LLYDANYFL	9	SB	E
KLWAQCVQL	9	SB	E
YLQPRTFLL	9	SB	E
ALWEIQQVV	9	SB	E
RLQSLQTYV	9	SB	E
LLLDRLNQL	9	SB	E
ILFTRFFYV	9	SB	E
YLYALVYFL	9	SB	E
ALSKGVHFV	9	SB	E
FIAGLIAIV	9	SB	E
FLNGSCGSV	9	WB	E
KIADYNYKL	9	SB	E
LLFNKVTLA	9	SB	E
FGDDTVIEV	9	SB	E
VLAWLYAAV	9	WB	E
FLLPSLATV	9	SB	E
FLHVTYVPA	9	WB	E
RLDKVEAEV	9	SB	E
FLFLTWICL	9		E
KLDDKDPNF	9	WB	
GMSRIGMEV	9	SB	E
LALLLLDRL	9		
KIYSKHTPI	9	WB	E
GLTVLPPLL	9	SB	E
ALNTLVKQL	9	WB	E
LITGRLQSL	9		
RLNEVAKNL	9	SB	
VLNDILSRL	9	SB	E
LLLLDRLNQL	10	WB	
KLPDDFTGCV	10	SB	
RLITGRLQSL	10	WB	
KLNDLCFTNV	10	SB	
YLGTGPEAGL	10	WB	

* Binary results obtained from the NetMHCpan-4.0 web server under the default threshold. SB and WB are short for strong binding and weak binding, respectively. All SB and WB epitopes are regarded as T-cell epitopes. ** Binary results obtained from the NetCTL-1.2 web server under the default threshold. E means NetCTL-1.2 recognized this T-cell epitope.
